# Activation of the coagulation cascade as a mechanism for selective nanoparticle-mediated RNA delivery to the endothelium in vivo

**DOI:** 10.1126/sciadv.ady2738

**Published:** 2025-10-22

**Authors:** Edward B. Guzman, Piotr S. Kowalski, Robert S. Langer, Daniel G. Anderson

**Affiliations:** ^1^ School of Engineering and Applied Sciences, Harvard University, Cambridge, MA, USA.; ^2^ David H. Koch Institute for Integrative Cancer Research, Massachusetts Institute of Technology, Cambridge, MA, USA.; ^3^ School of Pharmacy, University College Cork, Cork T12 K8AF, Ireland.; ^4^ APC Microbiome Ireland, University College Cork, Cork T12 K8AF, Ireland.; ^5^ Department of Chemical Engineering, Massachusetts Institute of Technology, Cambridge, MA, USA.; ^6^ Harvard and MIT Division of Health Sciences and Technology, Massachusetts Institute of Technology, Cambridge, MA, USA.; ^7^ Institute for Medical Engineering and Science, Massachusetts Institute of Technology, Cambridge, MA, USA.

## Abstract

Nanoparticles formulated with certain cationic lipids or polymers have been shown to facilitate RNA delivery to the endothelium. Here, we show that these nanoparticles become coated with coagulating proteins and induce coagulation for RNA delivery in vivo, without leading to histological evidence of clot formation. We further show that nanoparticles previously reported to transfect the liver can be redirected to other organs by preincubating them with procoagulant proteins. Our data demonstrate that activation of the coagulation cascade mediates endothelial RNA delivery and indicate that nanoparticles can be targeted to the endothelium of various organs by integrating procoagulant components in their formulation.

## Teaser

Nanoparticles formulated with procoagulant components will enable RNA delivery to endothelial cells of various organs.

## INTRODUCTION

The lack of RNA delivery systems to nonhepatic cells has been a barrier to the broad use of RNA therapy. There are a few rational strategies for cell targeting and limited knowledge about how existing nanoparticles reach cells outside of the liver. Nanoparticles formulated with cationic lipids or polymers can deliver RNA to endothelial cells with undetectable transfection of hepatocytes in vivo (*1*). For example, cationic lipids can be introduced in the formulation of RNA delivering nanoparticles to shift transfection from hepatocytes to lung endothelial cells (*2*, *3*). Optimizing the lipid ratios of such nanoparticles can further enhance selectivity for the lung endothelium (*4*). In other cases, conjugation of alkyl chains to cationic polymers produces lipid-polymer hybrids that form nanoparticles for selectively RNA delivery to endothelial cells in vivo (*5*, *6*).

The in vivo mechanism driving endothelial RNA delivery by cationic nanoparticles remains unclear, and we hypothesize that its definition could facilitate the design of RNA delivery systems. Several studies have suggested that plasma proteins are involved in nanoparticle targeting, with the formation of a protein corona on the nanoparticle surface enabling interactions with specific cell receptors (*7*–*15*). Among the proteins reported to facilitate endothelial targeting are vitronectin and fibrinogen (*14*, *16*, *17*)—two proteins known to regulate clot formation (*18*, *19*). Nanoparticles formulated with cationic lipids or polymers can also interact with negatively charged blood components, potentially forming microaggregates through ionic interactions or clotting processes. These aggregates may facilitate the passive accumulation of nanoparticles in capillaries, offering an alternative mechanism for endothelial targeting in vivo (*20*–*24*).

In this study, we demonstrate that nanoparticles formulated with cationic lipids or polymers are preferentially enriched with coagulation-related proteins in their protein corona and activate the coagulation cascade—a mechanism we find crucial in enabling RNA delivery to lung endothelial cells. We performed histological analysis of lung tissues and did not observe clot aggregates, suggesting that the nanoparticles may be cleared by pulmonary endothelial cells to prevent vessel occlusion. These findings indicate the importance of coagulation processes in functional RNA delivery, but they do not support the notion that microaggregates are trapped in capillaries to facilitate RNA delivery.

We further demonstrate that administration of the nanoparticles into the portal vein enabled partial transfection of liver endothelial cells and that in vivo knockout of key proteins involved in endocytosis or lipid metabolism did not affect endothelial transfection. Our data therefore suggest that nanoparticle uptake is driven by an anticoagulant pathway used by the entire endothelium. Building on these findings, we introduce a strategy to redirect nanoparticles—previously reported to transfect the liver—toward the lungs and other extrahepatic organs. This approach involves preincubating nanoparticles with procoagulant proteins to deliberately activate the coagulation cascade, thereby enabling RNA delivery to nonhepatic tissues. These results offer important insights into the mechanisms driving RNA delivery to endothelial cells in vivo, contributing to the development of nanoformulations for selective endothelial transfection.

## RESULTS

### Procoagulant proteins preferentially bind to endothelial nanoparticles

Plasma proteins have been shown to dictate nanoparticle tropism via the formation of a protein corona (*9*–*15*). A range of proteins, including fibrinogen, vitronectin, albumin, and various apolipoproteins, is commonly found in the corona of RNA delivery nanoparticles targeting the lung endothelium (*16*, *17*). From these proteins, albumin, apolipoprotein A (apoA), and apolipoprotein E (apoE) are typically among the most abundant, yet knockout studies in mice show that their absence does not impair RNA delivery to lung endothelial cells (*16*) (fig. S1). This suggests that other proteins, potentially less abundant, may be playing a more critical role in nanoparticle targeting. Moreover, the overlapping presence of similar proteins across chemically distinct nanoparticles complicates the task of identifying the specific proteins responsible for selective organ targeting (*16*, *17*, *25*), necessitating a more nuanced analysis of the protein corona to pinpoint key drivers of tissue tropism.

**Fig. 1.**
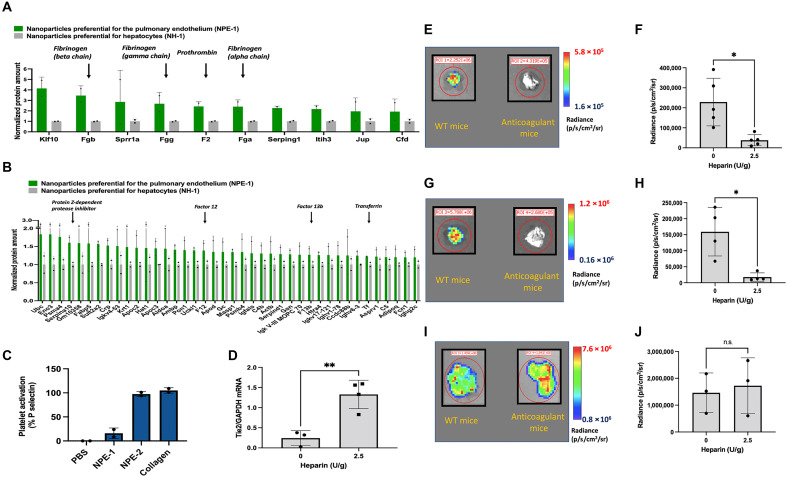
Nanoparticles formulated with cationic polymers or lipids preferentially associate with coagulating proteins to induce platelet activation and coagulation for pulmonary RNA delivery. (**A** and **B**) Normalized protein abundance from the corona of NPE-1 nanoparticles relative to the corona proteins from NH-1 nanoparticles. (A) Top 10 most preferential proteins to NPE-1. (B) Top 50 most preferential proteins to NPE-1, excluding the top 10 proteins already presented in (A). (**C**) Amount of platelet activation induced by NPE nanoparticles. Collagen was used as a positive control, and PBS alone was added in place of nanoparticles to get the baseline of P-selectin expression. (**D**) Tie2 knockdown in wild-type mice [heparin (0 U/g)] or anticoagulant-treated mice [heparin (2.5 U/g)] by NPE-1 nanoparticles injected with siTie2 (0.15 mg/kg) intravenously. GAPDH, glyceraldehyde-3-phosphate dehydrogenase. (**E** and **F**) Luciferase expression and luminescence quantification from the lungs of wild-type mice [heparin (0 U/g)] or anticoagulant-treated mice [heparin (2.5 U/g)] 6 hours after intravenous injection of NPE-3 nanoparticles encapsulating luciferase mRNA. Nanoparticles were injected with mRNA (1 mg/kg). (**G** and **H**) Luciferase expression and luminescence quantification from the lungs of wild-type mice [heparin (0 U/g)] or anticoagulant-treated mice [heparin (2.5 U/g)] 6 hours after intravenous injection of NPE-4 nanoparticles formulated with luciferase mRNA. Nanoparticles were injected with mRNA (0.1 mg/kg). (**I** and **J**) Luciferase expression and luminescence quantification in the liver of wild-type mice [heparin (0 U/g)] or anticoagulant-treated mice [heparin (2.5 U/g)] 6 hours after intravenous injection of NPE-4 nanoparticles formulated with luciferase mRNA. Nanoparticles were injected with mRNA (0.1 mg/kg). Data are shown as means ± SD. *N* = 2 to 5 per group. Biological replicates shown. *P < 0.05; **P < 0.005; n.s., not statistically significant, using a two-tailed, unpaired *t* test.

To better understand the proteins involved in organ-specific targeting, we conducted a comparative analysis of the protein coronas on nanoparticles with different tissue tropisms. For this, we first identified and quantified proteins in the coronas of nanoparticles preferentially targeting the pulmonary endothelium (NPE) and nanoparticles preferential for hepatocytes (NH), as described in the Materials and Methods section. The chemical composition and physicochemical properties of these nanoparticles can be seen in Table 1 and table S1. We then compared the abundance of each individual protein identified from the corona of NPE nanoparticles to their respective abundance on NH nanoparticles, allowing us to identify proteins that are preferential to endothelial-targeting nanoparticles rather than the most enriched overall (Fig. 1, A and B).

**Table 1.** Nanoparticle formulations. PEG, polyethylene glycol.

Nanoparticle preferential to pulmonary endothelium (NPE)	Composition	Mass %	Mole %	RNA type	Ionizable lipid or cationic polymer to RNA wt ratio
NPE-1	7C1:PEG	85:15	–	siRNA	5
NPE-2	C12-200:DOPE:DOTAP:cholesterol:C14-PEG-2000	–	17.5:8:50:23.25:1.25	mRNA	10
NPE-3	7C1:PEG	85:15	–	mRNA	5
NPE-4	C12-200:DOPE:DOTAP:cholesterol:C14-PEG-2000	–	30.63:14:12.5:40.69:2.19	mRNA	10
NPE-5	C12-200:DOPE:DOTAP:cholesterol: C14-PEG-2000	–	26.25:12:25:34.88:1.88	mRNA	10
**Nanoparticle preferential to hepatocytes (NH)**	**Composition**	**Mass %**	**Mole %**	**RNA type**	**Ionizable lipid or cationic polymer to RNA wt ratio**
NH-1	C12-200:DSPC:cholesterol:C14-PEG-2000	–	50:10:38.5:1.5	siRNA	5
NH-2	C12-200:DOPE:cholesterol:C14-PEG-2000	–	35:16:46.5:2.5	mRNA	10
NH-3	DLin-MC3-DMA:DOPE:cholesterol:C14-PEG-2000	–	35:16:46.5:2.5	mRNA	10
NH-4	cKK-E12:DOPE:cholesterol:C14-PEG-2000	–	35:16:46.5:2.5	mRNA	10

Our results showed that fibrinogen—including all three of its subunits (alpha, beta, and gamma)—as well as prothrombin, were among the top 10 proteins with the highest binding affinity for NPE nanoparticles (Fig. 1A). Fibrinogen is a key protein involved in coagulation, cellular adhesion, inflammation, and wound healing (*26*). This protein is functionally linked to prothrombin in a procoagulant environment, where prothrombin is converted into its active form, thrombin (*27*). Thrombin cleaves fibrinogen to expose the arginyl-glycyl-aspartate (RGD) motif, which can bind to integrin receptors expressed on endothelial cells, enabling targeted endothelial interactions (*28*). On the basis of this, we hypothesized that a procoagulant microenvironment forms on the surface of NPE nanoparticles, inducing prothrombin activation and subsequent fibrinogen cleavage to promote endothelial targeting. To test this hypothesis, we investigated whether endogenous proteins known to contribute to a procoagulant state exhibit preferential binding to NPE nanoparticles.

Examination of the next 40 most preferential proteins to NPE nanoparticles reveals that additional proteins associated with coagulation were preferentially binding to NPE nanoparticles, namely, protein Z–dependent protease inhibitor, factor XII, factor XIIIb, and transferrin (Fig. 1B). Among these, protein Z–dependent protease inhibitor functions as an anticoagulant, regulating blood coagulation to prevent excessive clot formation and unlikely to promote binding to endothelial cells (*29*). However, factor XII, factor XIIIb, and transferrin contribute to procoagulant conditions in vivo, playing a potential role in nanoparticle targeting to the endothelium. Specifically, factor XII plays a key role in initiating the intrinsic pathway of the coagulation cascade—a pathway known to be activated by cationic nanoparticles (*22*, *24*). Factor XIIIb acts as the carrier for the catalytic subunit A of factor XIII, which can be cross-linked with fibrinogen to expose its RGD motifs for adhesion to endothelial cells (*30*, *31*). Transferrin, a multifunctional protein, has also been implicated in enhancing thrombin activity while inhibiting antithrombotic pathways, thereby promoting fibrinogen cleavage and subsequent endothelial adhesion (*32*). Together, these findings demonstrate that NPE nanoparticles foster a complex, procoagulant protein corona that likely induces endothelial targeting via RGD-integrin binding pathways (*30*, *33*).

NPE nanoparticles generally exhibit minimal to no transfection of hepatocytes, and evaluation of the least preferential proteins to NPE nanoparticles reveals that apolipoproteins, including apoE, had the lowest affinity to NPE in comparison to NH nanoparticles (fig. S2A). This finding aligns with prior research showing that apoE plays a key role in directing RNA-loaded nanoparticles to hepatocytes (*7*, *8*), offering a plausible explanation for the limited hepatocyte transfection seen with NPE nanoparticles (*3*, *5*). Notably, the preferential enrichment of procoagulant proteins on NPE nanoparticles proposes that coagulating proteins are critical for directing nanoparticles to the lung endothelium (Fig. 1, A and B).

In addition to the most preferentially enriched proteins on NPE nanoparticles, other proteins may contribute to endothelial targeting. One example is vitronectin, which is a multifunctional protein involved in complement regulation (*34*), neural function (*35*), blood vessel formation (*36*), and wound healing (*37*). This protein has been detected on the surface of NPE nanoparticles that, like fibrinogen, contains an RGD domain that mediates binding to integrins on endothelial cells (*38*). However, our results show that vitronectin is equally abundant on both NPE and NH nanoparticles, suggesting that this protein may not play a major role in targeting of the lung endothelium, which is in congruence with other reports (*17*, *39*) (fig. S2B). Investigating the behavior of the nanoparticles in vitronectin knockout mice could clarify any potential role this protein might play in endothelial targeting. This type of evaluation is particularly important for certain endothelial nanoparticles smaller than 80 nm as fibrinogen may have minimal binding to these particles (*24*), potentially allowing other RGD-containing proteins to facilitate endothelial targeting (*38*). Similarly, the binding orientation of vitronectin and other RGD-containing proteins should be assessed to determine how exposure of RGD motifs affects endothelial targeting.

### Activation of the coagulation cascade enables endothelial RNA delivery

Certain nanoparticles formulated with cationic lipids and polymers have shown to induce platelet activation through the intrinsic coagulation pathway (*21*, *22*, *24*, *40*). The extent of platelet activation and coagulation correlates with the amount of cationic lipids used in the formulation (*22*), which coincidentally correlates with the transfection efficiency of nanoparticles in lung endothelial cells (*3*). Our proteomic analysis revealed that coagulation proteins preferentially bind to endothelial nanoparticles, making us believe that platelet activation could play a critical role in the transfection of lung endothelial cells. To test this, we evaluated whether nanoparticles could induce platelet activation and whether this activation is necessary for RNA delivery to pulmonary endothelial cells. NPE-1 and NPE-2 nanoparticles, formulated as described in Table 1 and having the physicochemical properties shown in table S1, were incubated with platelet-rich plasma for 30 min to then measure P-selectin expression, a common marker of platelet activation (*21*, *41*, *42*). Both NPE-1 and NPE-2 nanoparticles activated platelets, with NPE-2 showing higher platelet activation likely due to its higher zeta potential, reaching levels similar to those induced by collagen, a strong platelet activator (*43*) (Fig. 1C). NH nanoparticles had undetectable levels of platelet activation. These findings align with previous studies indicating that nanoparticles containing cationic lipids or polymers activate platelets (*21*, *22*, *24*).

Having found that NPE nanoparticles induce platelet activation and preferentially associate with coagulation proteins, we assessed pulmonary transfection in anticoagulant-treated mice to determine whether activation of the coagulation cascade along with platelet activity is critical for in vivo RNA delivery to the lung endothelium. For this experiment, we formulated two types of nanoparticles, NPE-1 and NPE-3, which share a similar chemical composition but differ in RNA cargo [small interfering RNA (siRNA) versus messenger RNA (mRNA); see Table 1]. Mice were pretreated with heparin anticoagulant to inhibit coagulation, 1 hour before the intravenous administration of nanoparticles.

Transfection analysis of the lungs revealed that both NPE-1 and NPE-3 nanoparticles lost their ability to transfect pulmonary endothelial cells in heparin-treated mice, indicating that activation of the coagulation cascade is essential for both siRNA and mRNA delivery to the lung endothelium (Fig. 1, D and F). In contrast, no changes in transfection were observed in the liver, heart, or kidneys of anticoagulant-treated mice relative to wild-type mice, suggesting that anticoagulant treatment does not induce a new tropism (fig. S3A).

Next, we repeated the experiment using NPE-4 nanoparticles, which contain a 12.5% concentration of 1,2-dioleoyl-3-trimethylammonium-propane (DOTAP), designed to enable concurrent transfection of lung endothelial cells and liver hepatocytes (*3*) (Table 1). In this case, transfection was absent in the lungs of heparin-treated mice but persisted in the liver as compared to wild-type controls (Fig. 1, G to J). This finding further demonstrates that activation of the coagulation cascade is required for transfection of the lung endothelium but not liver hepatocytes. No transfection was observed in the heart or kidneys of either wild-type or anticoagulant-treated mice with NPE-4 nanoparticles (fig. S3B). The presence of heparin in the bloodstream did not affect nanoparticle stability, as evidenced by the unchanged liver transfection, suggesting that heparin does not directly interact with the nanoparticles in vivo (Fig. 1, I and J).

The coagulation cascade is a complex system involving various clotting factors that ultimately lead to thrombin activation. Heparin functions by binding to antithrombin, which inactivates thrombin as well as other coagulation factors, including factor X (*44*). This broad mechanism gives heparin multiple pathways to inhibit clotting. Research has shown that bivalirudin—a direct thrombin inhibitor—does not affect nanoparticle transfection in the lungs (*24*). This effect might be due to bivalirudin’s mechanism of action, which blocks thrombin’s active site without inhibiting the activation of other coagulation factors in the blood (*45*). As such, nanoparticles can transfect endothelial cells in the presence of bivalirudin as other coagulating enzymes besides thrombin remain activated in the circulation. Gaining a deeper understanding of how endothelial cells internalize nanoparticles could provide further insights into the relationship between the coagulation cascade and transfection of the endothelium.

It is well known that coagulation within blood vessels can lead to vessel occlusion, resulting in serious side effects (*46*). To assess the potential for such complications, we administered the nanoparticles to mice at a dose that was 10 times higher than the dosage required for efficient pulmonary transfection. We then examined the lungs for signs of an embolism or blood flow obstructions at various time points when microaggregates have been detected inside of blood vessels due to nanoparticle administration (*20*).

**Fig. 2.**
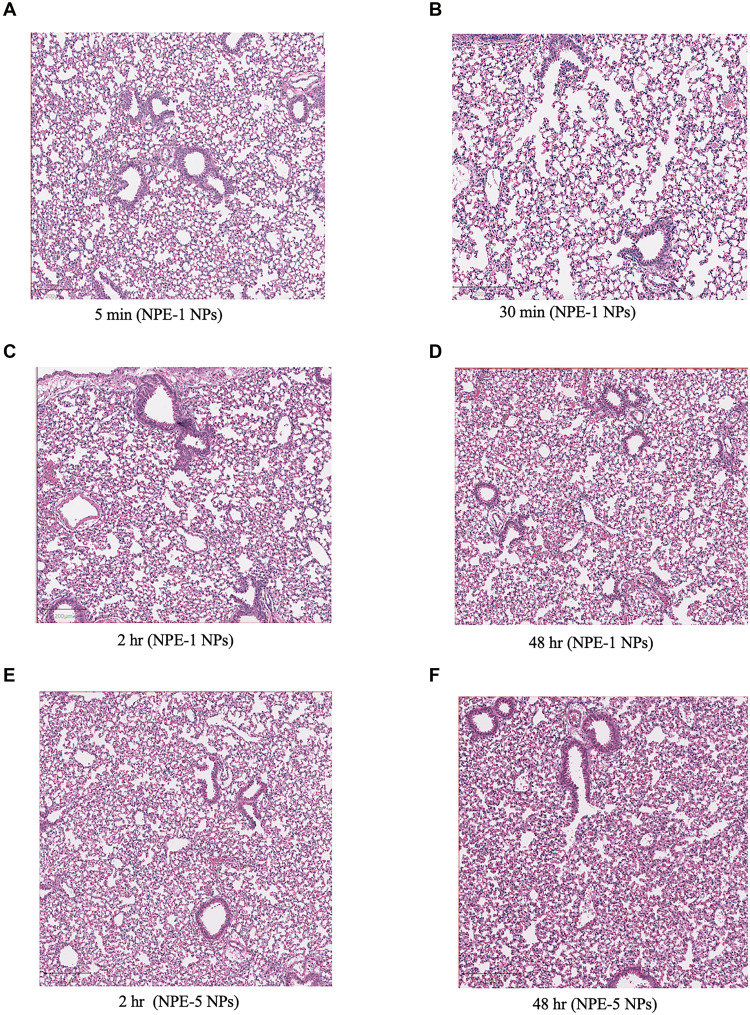
Representative posttreatment histology images from wild-type mice treated with endothelial nanoparticles at 10x the dose needed for effective transfection. Hematoxylin and eosin were used to stain the tissue. No signs of vessel occlusion or clots were observed during a 48-hour time period after nanoparticle administration. (**A** to **D**) Lung from mice treated with NPE-1 nanoparticles at the indicated time points postadministration of the NPs at a dose of 1 mg/kg of siRNA. (**E** and **F**) Lung from mice treated with NPE-5 nanoparticles at the indicated time points postadministration of the NPs at a dose of 1 mg/kg of mRNA. Biological replicate shown. *N* = 2 mice per group. NPs, nanoparticles; hr, hours.

Histological analysis of lung tissues from mice treated with nanoparticles formulated with either siRNA or mRNA revealed no noticeable aggregates or vessel occlusion when stained with hematoxylin and eosin (H&E). The lung tissues appeared similar to the lungs from mice treated with phosphate-buffered saline (PBS) or nanoparticles targeting hepatocytes at the same dose (Fig. 2, A to F, and fig. S4, A to D). The same results were observed with Carstair staining, which labels key coagulating components (fig. S5, A and B). The nanoparticle size also remained within the nanometer range after exposure to platelet-poor plasma, consistent with published results about the stability of cationic lung targeting nanoparticles in serum (*47*) (table S2). These results demonstrate that siRNA or mRNA is delivered by the nanoparticles without inducing passive accumulation of microaggregates within pulmonary capillaries.

Although some studies have observed notable thrombosis within blood vessels, likely due to the nanoparticle lipid composition evaluated (*22*, *24*), our findings are consistent with previous research showing that endothelial-targeting nanoparticles do not induce gross lesions or major underlying pathologies in mice and nonhuman primates (*3*, *5*, *6*). Clots formed by some cationic nanoparticles are detected in the first capillary bed downstream of injection, such as in the lungs following an intravenous administration or in the brain after an intra-arterial injection (*24*). We did not observe overt clots in the lungs after intravenous administration of NPE nanoparticles. Prior reports have also shown that injection of NPE nanoparticles do not induce an aberrant vasculature in major organs, an indication that large clots are not formed inside blood vessels (*3*, *6*). Collectively, these findings support a mechanism by which endothelial cells might actively scavenge procoagulant nanoparticles from the circulation by interacting directly with fibrinogen or via the binding of platelets to the vascular wall (*17*, *23*).

### Transfection is preferential but not restricted to the lung endothelium

**Fig. 3.**
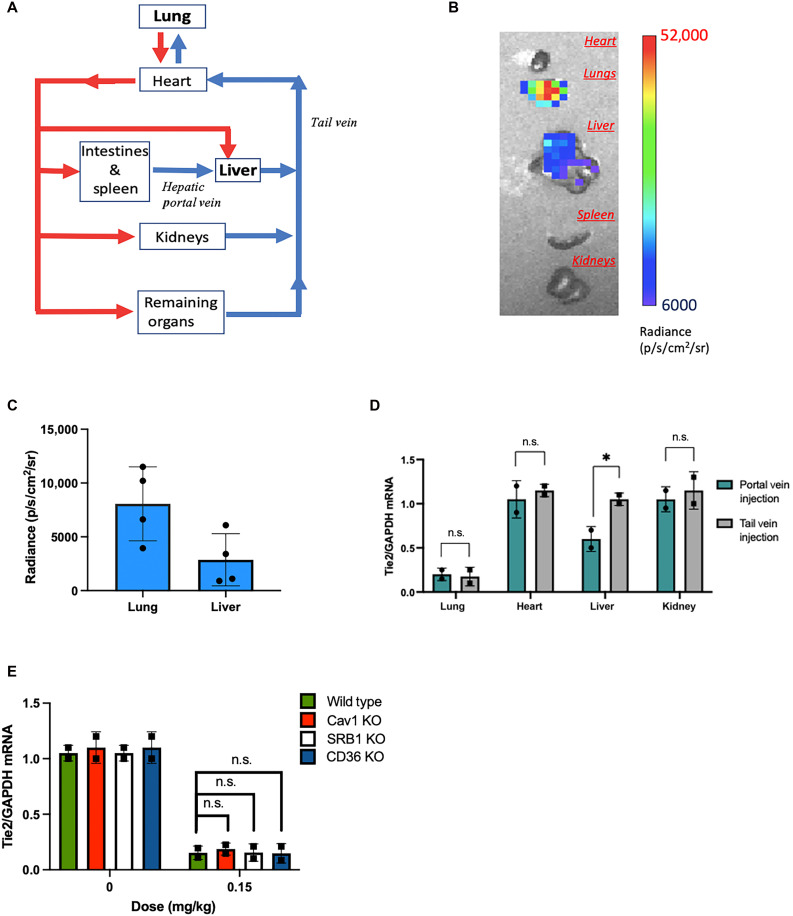
Transfection of endothelial nanoparticles when injected into the portal vein of wild-type mice or tail vein of knockout mice. (**A**) Schematic diagram of the blood flow, indicating the location of portal and tail veins with respect to the organs they perfuse. (**B**) Luciferase expression in organs of wild-type mice 24 hours after portal vein injection of NPE-2 nanoparticles loaded with luciferase mRNA (0.1 mg/kg). (**C**) Luminescence quantification in the lungs and liver 24 hours after portal vein injection of NPE-2 nanoparticles loaded with luciferase mRNA (0.1 mg/kg). (**D**) Tropism of endothelial nanoparticles after intravenous injection into the portal vein or tail vein. Tie2 knockdown in various organs of wild-type mice by NPE-1 nanoparticles 48 hours after nanoparticle intravenous administration into the portal vein or tail vein. Nanoparticles were formulated with siTie2 and injected with siRNA (0.15 mg/kg). (**E**) Tie2 knockdown in the lungs of Cav1, SRB1, or CD36 knockout (KO) and wild-type mice by NPE-1 nanoparticles 48 hours after nanoparticle administration into the tail vein. Nanoparticles were formulated with siTie2 and injected with siRNA (0.15 mg/kg). *N* = 2 to 4 per group. *P < 0.05; n.s., not statistically significant when *P* > 0.05, using a two-tailed, unpaired *t* test. Biological replicates shown.

The lung is one of the first organs encountered following a tail vein injection, containing the largest capillary network in the body and capable of accommodating the entire cardiac output (*48*–*50*). This led us to question whether nanoparticle selectivity for the lung is influenced by endothelial heterogeneity or whether it is the result of a nonselective first-passage effect. To explore this, we injected NPE-1 and NPE-2 into the portal vein, directing the nanoparticles straight into the liver vasculature, to determine whether the first-passage effect contributes to nanoparticle transfection. Portal vein injection ensures that the nanoparticles interact with hepatic endothelial cells first, rather than lung endothelial cells as in a tail vein administration (Fig. 3A). Our findings revealed that, whereas transfection was highest in the lung, lower transfection levels were also observed in the liver for both nanoparticle types (Fig. 3, B to D). This demonstrates that endothelial nanoparticles are not restricted to the pulmonary vasculature and that they are internalized through a mechanism shared by endothelial cells across various organs, with enhanced transfection in the lung due to its unique physiological characteristics (*50*). These results align with our previous works, which showed that NPE nanoparticles transfect the endothelium of various organs less efficiently than the lungs (*5*, *6*).

We next investigated membrane proteins implicated in endocytosis and lipid metabolism to better understand how nanoparticles bind to and are internalized by the endothelium. Caveolin-1 (Cav1) is a key membrane protein, especially abundant in the lung endothelium. It plays a critical role in caveolae formation in endothelial cells and in regulating endocytosis (*51*–*54*). Notably, Cav1 has been linked to the uptake of lipid nanoparticles by endothelial cells (*55*), prompting us to hypothesize that Cav1 could be a target for endothelial nanoparticles. To test this hypothesis, we assessed endothelial transfection in Cav1-deficient mice to determine whether the absence of this protein would hinder RNA delivery. Our results showed that Cav1 is not essential for nanoparticle uptake as endothelial transfection in Cav1 knockout mice was similar to that as in wild-type mice (Fig. 3E).

We also considered class B scavenger receptors, which are present on endothelial cell membranes and are known to facilitate the transport of lipoproteins, cholesterol, and fatty acids across the endothelium (*56*, *57*). Given that endothelial nanoparticles are formulated with lipids, lipid-like polymers, or even cholesterol to achieve optimal pulmonary transfection (*5*), we hypothesized that class B scavenger receptors—specifically SR-B1 and CD36—could be involved in the targeting mechanism of these nanoparticles. As with Cav1, we evaluated endothelial transfection in mice deficient in SR-B1 or CD36 to assess the role of these proteins in RNA delivery. The results revealed that the absence of either SR-B1 or CD36 had no impact on transfection efficacy, indicating that these proteins are not essential for RNA delivery to the pulmonary endothelium (Fig. 3E).

Other classes of endothelial receptors may be driving nanoparticle uptake, particularly those involved in scavenging procoagulant proteins or activated platelets from the circulation. Examples include thrombomodulin, endothelial integrins, and low-density lipoprotein receptor–related protein (LRP). These receptors are likely involved in the uptake of the nanoparticles by endothelial cells given their crucial role in regulating blood flow (*1*, *58*, *59*) and the ability of NPE nanoparticles to initiate coagulation for RNA delivery (Fig. 1).

### Purposely inducing coagulation to transfect various organs

**Fig. 4.**
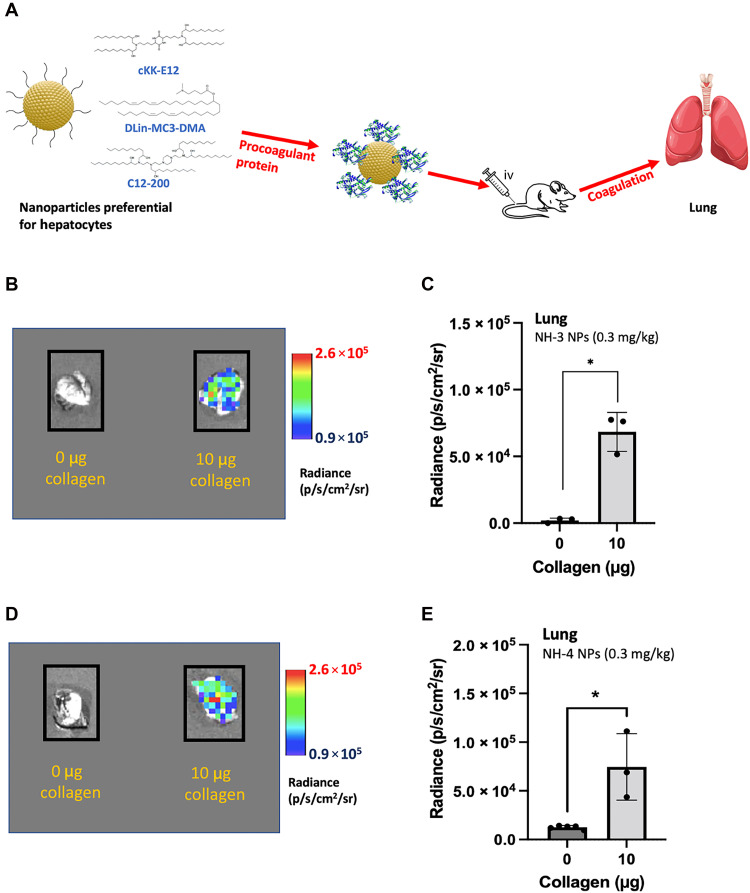
Liver-targeted nanoparticles can be directed to the lungs by preincubating them with a procoagulant protein. (**A**) NH-2, NH-3, and NH-4 nanoparticles containing the core lipid C12-200, DLin-MC3-DMA, and cKK-E12, respectively, were preincubated with distinct procoagulant proteins before intravenous (iv) administration. (**B** and **C**) NH-3 nanoparticles can transfect the lungs by preincubating them with procoagulant collagen. Luciferase mRNA was encapsulated in NH-3 nanoparticles, and lung transfection was evaluated by measuring luminescence 6 hours after injection. Nanoparticles were injected intravenously with RNA (0.3 mg/kg) containing 0 or 10 μg of collagen in PBS for each injection. (**D** and **E**) Transfection in the lung of mice treated with NH-4 nanoparticles preincubated with 0 or 10 μg of collagen. Nanoparticles were formulated with luciferase mRNA, and transfection was evaluated by measuring luminescence. Each mice received an intravenous injection of NH-4 nanoparticles containing 0 or 10 μg of collagen with RNA (0.3 mg/kg). Transfection was assessed 6 hours postinjection. Data are shown as means ± SD. *N* = 3 to 6 mice per group. *P < 0.05, using a two-tailed, unpaired *t* test. Biological replicates shown.

**Fig. 5.**
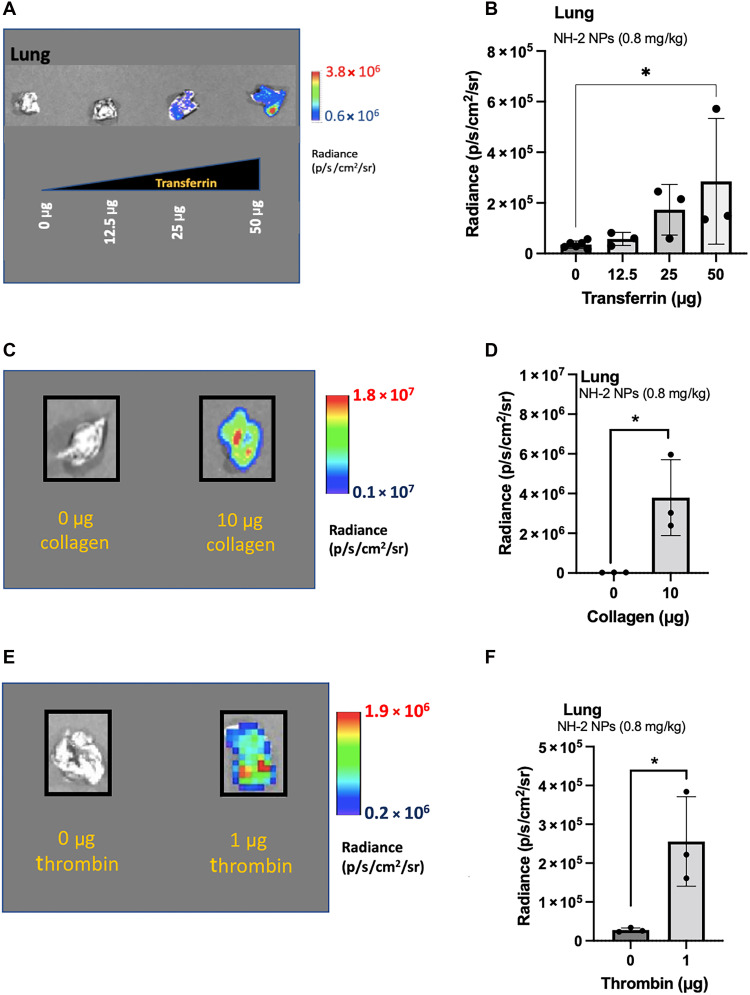
Distinct procoagulant proteins can be used to direct liver-targeted nanoparticles to the lung. (**A**) NH-2 nanoparticles were preincubated for 30 min with various concentrations of transferrin to subsequently inject them intravenously for RNA delivery to the lungs. Luciferase expression of NH-2 nanoparticles encapsulating luciferase mRNA was measured in the lungs 6 hours after intravenous injection of the nanoparticles. The nanoparticles were injected with mRNA (0.8 mg/kg) containing the indicated amount of transferrin. (**B**) Luminescence quantification in the lungs of mice treated with NH-2 nanoparticles with various amounts of transferrin. Nanoparticles were injected with mRNA (0.8 mg/kg) and the specified amount of transferrin. (**C** and **D**) NH-2 nanoparticles formulated with luciferase mRNA and preincubated for 30 min with collagen before intravenous injection for lung transfection. Luciferase expression was evaluated 6 hours after intravenous injection. Nanoparticles were injected with mRNA (0.8 mg/kg) and 0 or 10 μg of collagen. (**E**) Preincubation of NH-2 nanoparticles with thrombin for 30 min before intravenous injection for pulmonary transfection. Nanoparticles were formed with luciferase mRNA, and transfection, shown in the form of luminescence, was evaluated 6 hours after intravenous injection. NH-2 nanoparticles were injected with mRNA (0.8 mg/kg) and 0 or 1 μg of thrombin. (**F**) Luminescence quantification from mice treated with NH-2 nanoparticles, demonstrating that lung transfection is possible by adding procoagulant thrombin to the formulation of NH-2. Data are shown as means ± SD. *N* = 3 to 6 mice per group. *P < 0.05, using a two-tailed, unpaired *t* test. Biological replicates shown.

Cationic lipids are notorious for activating platelets and the coagulation cascade (*21*, *22*), and addition of such lipids into nanoparticle formulations enables transfection of the lung (*2*, *3*). On the basis of this, we hypothesized that substituting cationic lipids with procoagulant proteins could similarly promote pulmonary transfection through the activation of the coagulation cascade. To test this, we formulated nanoparticles preferential for hepatocytes, namely, NH-2, NH-3, and NH-4, which contained the ionizable lipids C12-200 (*60*), MC3 (*61*), and cKK-E12 (*8*), respectively (Table 1). We then incubated these nanoparticles with procoagulant proteins to assess their potential for lung transfection (Fig. 4A). Collagen was selected as the procoagulant protein due to its well-characterized role in platelet activation and coagulation (*43*). Nanoparticles were incubated with collagen for 30 min before being injected intravenously, and pulmonary transfection was evaluated 6 hours postinjection. Results showed that all nanoparticles induced lung transfection, confirming that collagen addition to NH nanoparticles directs them to the lung (Figs. 4, B and E, and 5, C and D). Notably, the ionizable lipid type in the nanoparticle formulation did not affect lung transfection when collagen was present, unlike the liver where lipid type affects efficiency (fig. S6, A and B). This demonstrates that pulmonary RNA delivery using collagen can be achieved with a variety of ionizable lipids without compromising efficacy.

Building on the observation that collagen directed NH nanoparticles to the lung, we tested additional procoagulant proteins to determine whether pulmonary transfection can be achieved with other procoagulant proteins. This time we selected thrombin and transferrin, both of which are procoagulants proteins and expected to induce coagulation and platelet activation in combination with the nanoparticles upon entering the circulation (*32*, *62*).

As with collagen, NH-2 nanoparticles encapsulating luciferase mRNA were incubated with either thrombin or transferrin for 30 min and then intravenously administered. Pulmonary transfection was assessed 6 hours postinjection. Our results showed that NH-2 nanoparticles successfully transfected the lung when combined with either thrombin or transferrin, demonstrating that pulmonary RNA delivery is achievable with distinct procoagulant proteins (Fig. 5, A, B, E, and F).

The level of protein preincubated with the nanoparticles directly correlated with the transfection efficiency in the lung, reflecting coagulation levels proportional to each protein concentration (*32*) (Fig. 5, A and B). Similar transfection levels were observed with collagen and thrombin, with thrombin requiring lower protein amounts due to its strong coagulation potency (*63*) (fig. S6C). These findings indicate that the efficiency of pulmonary RNA delivery systems can be enhanced by promoting stronger platelet activation and coagulation. Endothelial transfection was detected with the procoagulant proteins and the nanoparticles maintained their stability during the preincubation period, as their size remained within the nanometer range before injection (fig. S6D, table S3).

We then wanted to see whether transfection was possible in additional organs besides the lungs by preincubating NH nanoparticles with procoagulant proteins. We selected collagen again as our procoagulant protein and preincubated NH-2 nanoparticles with this protein for 30 min before intravenous injection. Although not statistically significant, transfection in the kidney was observed through this method, supporting our finding that the coagulation mechanism is preferential but not restrictive to the lungs (fig. S7, A and B). In the liver and spleen, a slight increase in transfection was noted, likely due to the uptake of the nanoparticles by endothelial cells in these tissues; however, this effect was also not statistically significant (fig. S7, C to F). These data demonstrate that nanoparticles can be directed to multiple organs via the coagulation mechanism and highlight the versatility of the nanoparticles at transfecting a wide range of tissues. Of note, injection of the nanoparticles with procoagulant proteins does lead to signs of vessel occlusion and inflammation, likely due to the presence of unbound proteins not fully attached to the nanoparticle surface (fig. S8). Enhancing the binding affinity between the procoagulant proteins and the nanoparticles, potentially thought direct conjugation (*7*), may allow for a lower protein dose needed to minimize side effects. Such an approach could also pave the way for the development of RNA conjugates, with the procoagulant protein acting as a targeting moiety for delivery to extrahepatic tissues (*64*).

## DISCUSSION

Nanoparticles formulated with cationic lipids or polymers have been widely used for RNA delivery to pulmonary endothelial cells. However, the mechanism by which these nanoparticles facilitate RNA delivery remains largely unknown. In this study, we demonstrated that cationic nanoparticles require coagulation for effective RNA delivery to the lung endothelium, although they do not induce observable clot formation. In addition, we showed that cationic lipids can be substituted with procoagulant proteins for successful pulmonary transfection.

Our findings indicate that RNA delivery systems can be rationally designed using procoagulant materials to target the endothelium of various organs, particularly the lungs. However, the safety and tolerability of procoagulant RNA carriers remain a crucial area for future investigation, as pronounced coagulation within blood vessels could lead to adverse side effects (*46*). Previous studies have shown that some delivery nanoparticles containing cationic lipids or polymers do not induce gross lesions or major pathologies in mice and nonhuman primates, indicating that the level of coagulation they cause is likely well tolerated (*3*, *5*, *6*). Nevertheless, these studies were conducted in healthy animals, so caution should be exercised when using these delivery systems in procoagulant diseases (*24*).

We hypothesize that antithrombotic receptors, ubiquitously expressed on endothelial cells, may scavenge procoagulant nanoparticles from the circulation to facilitate transfection. This is particularly plausible given that our results show that endothelial transfection is not limited to the lungs and that anticoagulant receptors are present throughout the vascular endothelium (*65*). Despite these insights, noncoagulating RNA delivery mechanisms will be required for patients on anticoagulant therapy. Our findings demonstrate that cationic nanoparticles are unable to transfect the lung endothelium in the presence of heparin, underscoring a limitation of procoagulant-based RNA delivery systems. Alternative strategies, such as noncoagulating nanoparticles coated with targeting ligands or endogenous extracellular vesicles, could offer viable options for RNA delivery in such patients (*66*–*68*).

## MATERIALS AND METHODS

Lipids were purchased from Avanti Polar Lipids Inc. MC3 was purchased from Organix Inc. 7C1 and C12-200 were synthesized through our lab according to (*5*, *60*). Proteins were purchased from Abcam or R&D Systems Inc. siRNA was synthesized by Integrated DNA Technologies, whereas the luciferase mRNA was kindly donated by Translate Bio.

### Experimental designs

#### Nanoparticle preparation

RNA-loaded nanoparticles were created by mixing an organic and aqueous solution using a microfluidic device as previously described (*69*). The organic solution contained the lipids and polymer(s), whereas the aqueous solution contained RNA as described in Table 1. All nanoparticles encapsulating mRNA were formulated with luciferase mRNA, whereas nanoparticles containing siRNA were generated with scrambled siRNA for in vitro assays and histology inspections. siTie2 was used for in vivo transfection studies.

#### Nanoparticle characterization

Nanoparticle size measurements were performed using dynamic light scattering (DLS). For all size measurements, nanoparticles were suspended in PBS. When nanoparticles were preincubated with individual procoagulant proteins, size measurements were done directly without removing unbounded proteins. However, when the nanoparticles were exposed to plasma, the unbound proteins were washed as described in the protein corona identification procedure and resuspended in PBS, and then nanoparticles were assessed via DLS. For zeta potential measurements, nanoparticles were diluted in water and assessed via electrophoretic light scattering (ELS).

#### Protein corona identification

Nanoparticles were formulated as stated above with the addition of 0.45% mass ratio of DiI (1,1′-dioctadecyl-3,3,3′,3′-tetramethylindocarbocyanine perchlorate) in place of a 0.45% mass ratio of 7C1 or C12-200 to enable nanoparticle visualization during the protein identification procedure. The procedure consisted of incubating the nanoparticles with 10% mouse plasma at room temperature for 15 min. To obtain plasma, blood was collected from mice via cardiac puncture. Blood was then placed into tubes containing EDTA and centrifuged to separate cells and get the plasma. The nanoparticles were then added to the top of a 0.05 M sucrose cushion inside a polycarbonate tube and ultracentrifuged for 1.5 hours at 80,000*g* using a swinging rotor at 4°C. The resulting pellet containing the nanoparticles was collected and ran through a 300k centrifuge filter to concentrate the nanoparticles, which were then heated to 95°C for 5 min in the presence of SDS 1.2x to detach and denature the proteins from the nanoparticle surface. The proteins were separated by molecular weight using a polyacrylamide gel, allowing the proteins to be ordered on the basis of their abundance with respect to molecular weight. The proteins were then processed via tandem mass tag (TMT) mass spectrometry at the Biopolymers & Proteomics Core of the Koch Institute at MIT for protein identification and quantification. The proteins from the NPE and NH nanoparticles were processed in parallel to ensure accurate, side-by-side comparison of protein abundance between the two samples. For TMT labeling, the peptides were labeled with the TMTpro 16plex Label Reagent set purchased from Thermo Fisher Scientific. Desalted peptides were dissolved in 100 μl of 100 mM triethylammonium bicarbonate (pH 8.5), and the TMTpro 16plex Reagent was dissolved in 20 μl of anhydrous acetonitrile. The solution containing peptides were mixed with the appropriate TMT reagent, vortexed, and incubated at room temperature for 1 hour. For mass spectrometry (MS) and high-performance liquid chromatography (HPLC), the TMT-labeled tryptic peptides were separated by reversed-phase HPLC (Thermo Fisher Scientific UltiMate 3000) using a Thermo Fisher Scientific PepMap RSLC C18 column (2-μm tip, 75 μm by 50 cm, PN #ES903) over a 140-min gradient before nanoelectrospray using an Orbitrap Exploris 480 mass spectrometer (Thermo Fisher Scientific). Solvent A was 0.1% formic acid in water, and solvent B was 0.1% formic acid in acetonitrile. The gradient conditions were 1% B (0 to 10 min at 300 nl/min), 1% B (10 to 15 min, 300 to 200 nl/min), 1 to 5% B (15 to 20 min, 200 nl/min), 5 to 25% B (20 to 104.8 min, 200 nl/min), 25 to 35% B (104.8 to 112 min, 200 nl/min), 35 to 80% B (112 to 115.5 min, 200 nl/min), 80% B (115.5 to 120 min, 200 nl/min), 80 to 100% B (120 to 120.1 min, 200 nl/min), and 100% B (120.1.1 to 140 min, 200 nl/min). The mass spectrometer was operated in a data-dependent mode. The parameters for the full-scan MS were conducted at a resolution of 60,000 across 450 to 1600 *m/z* (mass/charge ratio) and a maximum IT of 50 ms. The full MS scan was followed by tandem mass spectrometry for as many precursor ions in a 2-s cycle with an NCE of 36, a dynamic exclusion of 30 s, and a resolution of 45,000. A complete list of the corona proteins and their absolute nonrelative amount on each nanoparticle can be found in tables S4 and S5.

#### Nanoparticle-induced platelet activation assessment

Blood was obtained from C57BL/6 mice via cardiac puncture, citrated with 4% (w/v) sodium citrate, and then centrifuged at 200*g* for 20 min to obtain platelet-rich plasma. The plasma was then diluted to 15% using PBS, and nanoparticles at 150 ng of RNA/μl were then added to plasma at a 1:1 volume ratio. The solution was then mixed and incubated for 30 min at room temperature. Collagen at 10 μg/ml or PBS alone was added instead of nanoparticles as a positive or negative control, respectively. After incubation, platelets were fixed with 1% paraformaldehyde in staining buffer and activated platelets were fluorescently labeled with P-selectin antibody. Flow cytometry was used to quantify P-selectin expression.

#### In vivo transfection evaluation and histology

Unless otherwise specified, nanoparticles were injected intravenously into the tail vein of C57BL/6 mice or knockout mice and organs were collected 6 hours after injection of nanoparticles loaded with luciferase mRNA or 48 hours when siRNA was encapsulated in the nanoparticles. For experiments where transferrin, collagen, or thrombin was introduced in the nanoparticle formulation, the proteins were added to the nanoparticles suspended in PBS and incubated for 30 min before injection. To quantify luciferase expression, d-luciferin was injected intraperitoneally at 150 mg/kg 15 min before euthanizing the mice. This way, luciferin was able to react with the produced luciferase protein and produced luminescence, which was quantified with an IVIS Spectrum In Vivo Imaging System by PerkinElmer. To quantify Tie2 knockdown, organs were harvested, and RNA was extracted to quantify expression via reverse transcription quantitative polymerase chain reaction. For histological analysis, organs were collected at 5 min, 30 min, 2 hours, or 48 hours postintravenous administration of the nanoparticles or PBS into the tail vein. Organs were then immersed in 10% neutral-buffered formalin, incubated for 24 to 48 hours, and embedded in paraffin, sectioned, imaged, and analyzed by the Histology Core Facility of the Koch Institute at MIT. All animal procedures were approved by the Institutional Animal Care and Use Committee and were in accordance with local, state, and federal regulations (MIT protocol: 0418-025-21). Unless otherwise noted, experiments were done in 5- to 8-week-old C57BL/6 female mice (Charles River Laboratories, random selection).

#### Transfection evaluation in anticoagulant-treated mice

Mice were intraperitoneally injected with heparin (2.5 U/g) 30 min before intravenous injection of nanoparticles. Control mice were injected with PBS instead of heparin. Nanoparticles were injected intravenously.

#### Portal vein administration

SR Buprenorphine (1 mg/kg) was subcutaneously administered the morning before surgery. To clean and aseptically prepare the surgical site, the mice were anesthetized under continuous flow of 1 to 4% isoflurane with oxygen at 0.5 liters/min using a calibrated vaporizer. A shaver with size #40 clipper blade was then used to remove hair to reveal an area of about 3 cm by 3 cm on the ventral midline of the animal’s abdomen. The entire shaved area was then aseptically prepared with a minimum of three cycles of scrubbing with povidine (in an outward centrifugal direction from the center of the incision site when possible), followed by rinsing with 70% alcohol. A final skin paint with povidine was also applied. The surgical site was then draped with sterile disposable paper to exclude surrounding hair from touching the surgical site. Once the surgical site has been prepared, a small incision of ~2.5 cm was made in the upper right abdomen. The skin and muscle layers were cut, and a sterile tissue retractor was placed to keep the incision open. The intestines and pancreas were then made visible, and they were moved to the side to expose the portal vein. The needle was then inserted into the portal vein for nanoparticle administration. After injection, the syringe was slowly retrieved and pressure was applied to prevent any backflow. The intestines and pancreas were placed back into the abdomen, and the incision was closed using a layer of sutures.

#### Tie2 siRNA sequence

The Tie2 siRNA sequence used in the study was as follows: sense: GAAGAuGcAGuGAuuuAcAdTsdT and antisense: UGuAAAUcACUGcAUCUUCdTsdT.

Lowercase letters correspond to nucleotides modified with 2-*O*-methyl modifications.

### Statistical analysis

All statistical analyses were conducted in Prism 9 using its default two-tailed, unpaired *t* test.

## References

[1] G. W. Liu, E. B. Guzman, N. Menon, R. S. Langer, Lipid nanoparticles for nucleic acid delivery to endothelial cells. Pharm. Res. 40, 3–25 (2023). 36735106 10.1007/s11095-023-03471-7 PMC9897626

[2] R. Dorkin, “Development and mechanistic analysis of in vivo liposomal nanoparticle delivery of siRNA and mRNA,” thesis, Massachusetts Institute of Technology, Cambridge, MA (2016).

[3] Q. Cheng, T. Wei, L. Farbiak, L. T. Johnson, S. A. Dilliard, D. J. Siegwart, Selective organ targeting (SORT) nanoparticles for tissue-specific mRNA delivery and CRISPR-Cas gene editing. Nat. Nanotechnol. 15, 313–320 (2020). 32251383 10.1038/s41565-020-0669-6 PMC7735425

[4] T. Wei, Y. Sun, Q. Cheng, S. Chatterjee, Z. Traylor, L. T. Johnson, M. L. Coquelin, J. Wang, M. J. Torres, X. Lian, X. Wang, Y. Xiao, C. A. Hodges, D. J. Siegwart, Lung SORT LNPs enable precise homology-directed repair mediated CRISPR/Cas genome correction in cystic fibrosis models. Nat. Commun. 14, 7322 (2023). 37951948 10.1038/s41467-023-42948-2 PMC10640563

[5] J. E. Dahlman, C. Barnes, O. Khan, A. Thiriot, S. Jhunjunwala, T. E. Shaw, Y. Xing, H. B. Sager, G. Sahay, L. Speciner, A. Bader, R. L. Bogorad, H. Yin, T. Racie, Y. Dong, S. Jiang, D. Seedorf, A. Dave, K. S. Sandu, M. J. Webber, T. Novobrantseva, V. M. Ruda, A. K. R. Lytton-Jean, C. G. Levins, B. Kalish, D. K. Mudge, M. Perez, L. Abezgauz, P. Dutta, L. Smith, K. Charisse, M. W. Kieran, K. Fitzgerald, M. Nahrendorf, D. Danino, R. M. Tuder, U. H. von Andrian, A. Akinc, A. Schroeder, D. Panigrahy, V. Kotelianski, R. Langer, D. G. Anderson, In vivo endothelial siRNA delivery using polymeric nanoparticles with low molecular weight. Nat. Nanotechnol. 9, 648–655 (2014). 24813696 10.1038/nnano.2014.84 PMC4207430

[6] O. F. Khan, P. S. Kowalski, J. C. Doloff, J. K. Tsosie, V. Bakthavatchalu, C. B. Winn, J. Haupt, M. Jamiel, R. Langer, D. G. Anderson, Endothelial siRNA delivery in nonhuman primates using ionizable low-molecular weight polymeric nanoparticles. Sci. Adv. 4, eaar8409 (2018). 29963629 10.1126/sciadv.aar8409 PMC6021147

[7] A. Akinc, W. Querbes, S. De, J. Qin, M. Frank-Kamenetsky, K. N. Jayaprakash, M. Jayaraman, K. G. Rajeev, W. L. Cantley, J. R. Dorkin, J. S. Butler, L. Qin, T. Racie, A. Sprague, E. Fava, A. Zeigerer, M. J. Hope, M. Zerial, D. W. Sah, K. Fitzgerald, M. A. Tracy, M. Manoharan, V. Koteliansky, A. Fougerolles, M. A. Maier, Targeted delivery of RNAi therapeutics with endogenous and exogenous ligand-based mechanisms. Mol. Ther. 18, 1357–1364 (2010). 20461061 10.1038/mt.2010.85 PMC2911264

[8] Y. Dong, K. T. Love, J. R. Dorkin, S. Sirirungruang, Y. Zhang, D. Chen, R. L. Bogorad, H. Yin, Y. Chen, A. J. Vegas, C. A. Alabi, G. Sahay, K. T. Olejnik, W. Wang, A. Schroeder, A. K. Lytton-Jean, D. J. Siegwart, A. Akinc, C. Barnes, S. A. Barros, M. Carioto, K. Fitzgerald, J. Hettinger, V. Kumar, T. I. Novobrantseva, J. Qin, W. Querbes, V. Koteliansky, R. Langer, D. G. Anderson, Lipopeptide nanoparticles for potent and selective siRNA delivery in rodents and nonhuman primates. Proc. Natl. Acad. Sci. U.S.A. 111, 3955–3960 (2014). 24516150 10.1073/pnas.1322937111 PMC3964096

[9] V. Francia, R. M. Schiffelers, P. R. Cullis, D. Witzigmann, The biomolecular corona of lipid nanoparticles for gene therapy. Bioconjug. Chem. 31, 2046–2059 (2020). 32786370 10.1021/acs.bioconjchem.0c00366

[10] C. Ge, J. Tian, Y. Zhao, C. Chen, R. Zhou, Z. Chai, Towards understanding of nanoparticle-protein corona. Arch. Toxicol. 89, 519–539 (2015). 25637415 10.1007/s00204-015-1458-0

[11] F. S. M. Tekie, M. Hajiramezanali, P. Geramifar, M. Raoufi, R. Dinarvand, M. Soleimani, F. Atyabi, Controlling evolution of protein corona: A prosperous approach to improve chitosan-based nanoparticle biodistribution and half-life. Sci. Rep. 10, 9664 (2020). 32541900 10.1038/s41598-020-66572-y PMC7295777

[12] C. Corbo, R. Molinaro, A. Parodi, N. E. Toledano Furman, F. Salvatore, E. Tasciotti, The impact of nanoparticle protein corona on cytotoxicity, immunotoxicity and target drug delivery. Nanomedicine 11, 81–100 (2016). 26653875 10.2217/nnm.15.188 PMC4910943

[13] P. Aggarwal, J. B. Hall, C. B. McLeland, M. A. Dobrovolskaia, S. E. McNeil, Nanoparticle interaction with plasma proteins as it relates to particle biodistribution, biocompatibility and therapeutic efficacy. Adv. Drug. Deliv. Rev. 61, 428–437 (2009). 19376175 10.1016/j.addr.2009.03.009 PMC3683962

[14] D. Chen, S. Ganesh, W. Wang, A. Lupieri, M. Amiji, Role of vitronectin-rich protein corona on tumor-specific siRNA delivery and transfection with lipid nanoparticles. Nanomedicine 16, 535–551 (2021). 33683145 10.2217/nnm-2020-0428

[15] F. Giulimondi, L. Digiacomo, D. Pozzi, S. Palchetti, E. Vulpis, A. L. Capriotti, R. Z. Chiozzi, A. Lagana, H. Amenitsch, L. Masuelli, G. Peruzzi, M. Mahmoudi, I. Screpanti, A. Zingoni, G. Caracciolo, Interplay of protein corona and immune cells controls blood residency of liposomes. Nat. Commun. 10, 3686 (2019). 31417080 10.1038/s41467-019-11642-7 PMC6695391

[16] S. A. Dilliard, Q. Cheng, D. J. Siegwart, On the mechanism of tissue-specific mRNA delivery by selective organ targeting nanoparticles. Proc. Natl. Acad. Sci. U.S.A. 118, e2109256118 (2021). 34933999 10.1073/pnas.2109256118 PMC8719871

[17] M. Qiu, Y. Tang, J. Chen, R. Muriph, Z. Ye, C. Huang, J. Evans, E. P. Henske, Q. Xu, Lung-selective mRNA delivery of synthetic lipid nanoparticles for the treatment of pulmonary lymphangioleiomyomatosis. Proc. Natl. Acad. Sci. U.S.A. 119, e2116271119 (2022). 35173043 10.1073/pnas.2116271119 PMC8872770

[18] A. S. Wolberg, Fibrinogen and fibrin: Synthesis, structure, and function in health and disease. J. Thromb. Haemost. 21, 3005–3015 (2023). 37625698 10.1016/j.jtha.2023.08.014 PMC10592048

[19] T. J. Podor, S. Campbell, P. Chindemi, D. M. Foulon, D. H. Farrell, P. D. Walton, J. I. Weitz, C. B. Peterson, Incorporation of vitronectin into fibrin clots: Evidence for a binding interaction between vitronectin and γa/γ′ fibrinogen. J. Biol. Chem. 277, 7520–7528 (2002). 11744726 10.1074/jbc.M109677200

[20] K. Minami, K. Okamoto, K. Doi, K. Harano, E. Noiri, E. Nakamura, siRNA delivery targeting to the lung via agglutination-induced accumulation and clearance of cationic tetraamino fullerene. Sci. Rep. 4, 4916 (2014). 24814863 10.1038/srep04916 PMC4017229

[21] S. Novakowski, K. Jiang, G. Prakash, C. Kastrup, Delivery of mRNA to platelets using lipid nanoparticles. Sci. Rep. 9, 552 (2019). 30679556 10.1038/s41598-018-36910-2 PMC6345896

[22] X. Wu, H. Chen, C. Wu, J. Wang, S. Zhang, J. Gao, H. Wang, T. Sun, Y. G. Yang, Inhibition of intrinsic coagulation improves safety and tumor-targeted drug delivery of cationic solid lipid nanoparticles. Biomaterials 156, 77–87 (2018). 29190500 10.1016/j.biomaterials.2017.11.040

[23] P. Chollet, M. C. Favrot, A. Hurbin, J. L. Coll, Side-effects of a systemic injection of linear polyethylenimine-DNA complexes. J. Gene Med. 4, 84–91 (2002). 11828391 10.1002/jgm.237

[24] S. Omo-Lamai, M. E. Zamora, M. N. Patel, J. Wu, J. Nong, Z. Wang, A. Peshkova, A. Majumder, J. R. Melamed, L. S. Chase, E. O. Essien, D. Weissman, V. R. Muzykantov, O. A. Marcos-Contreras, J. W. Myerson, J. S. Brenner, Physicochemical targeting of lipid nanoparticles to the lungs induces clotting: Mechanisms and solutions. Adv. Mater. 36, e2312026 (2024). 38394670 10.1002/adma.202312026 PMC11209818

[25] S. Tenzer, D. Docter, J. Kuharev, A. Musyanovych, V. Fetz, R. Hecht, F. Schlenk, D. Fischer, K. Kiouptsi, C. Reinhardt, K. Landfester, H. Schild, M. Maskos, S. K. Knauer, R. H. Stauber, Rapid formation of plasma protein corona critically affects nanoparticle pathophysiology. Nat. Nanotechnol. 8, 772–781 (2013). 24056901 10.1038/nnano.2013.181

[26] M. Soleimanpour, S. S. Mirhaji, S. Jafari, H. Derakhshankhah, F. Mamashli, H. Nedaei, M. R. Karimi, H. Motasadizadeh, Y. Fatahi, A. Ghasemi, M. S. Nezamtaheri, M. Khajezade, M. Teimouri, B. Goliaei, C. Delattre, A. A. Saboury, Designing a new alginate-fibrinogen biomaterial composite hydrogel for wound healing. Sci. Rep. 12, 7213 (2022). 35508533 10.1038/s41598-022-11282-w PMC9068811

[27] S. Krishnaswamy, The transition of prothrombin to thrombin. J. Thromb. Haemost. 11, 265–276 (2013). 23809130 10.1111/jth.12217 PMC3713535

[28] Y. M. Stohnii, T. A. Yatsenko, V. V. Nikulina, Y. P. Kucheriavyi, O. O. Hrabovskyi, O. Y. Slominskyi, K. S. Savchenko, L. V. Garmanchuk, L. D. Varbanets, A. O. Tykhomyrov, V. O. Chernyshenko, Functional properties of individual sub-domains of the fibrin(ogen) αC-domains. BBA Adv. 3, 100072 (2023). 37082262 10.1016/j.bbadva.2023.100072 PMC10074951

[29] X. Huang, R. Swanson, H. K. Kroh, P. E. Bock, Protein Z-dependent protease inhibitor (ZPI) is a physiologically significant inhibitor of prothrombinase function. J. Biol. Chem. 294, 7644–7657 (2019). 30918026 10.1074/jbc.RA118.006787 PMC6514612

[30] A. M. Belkin, G. Tsurupa, E. Zemskov, Y. Veklich, J. W. Weisel, L. Medved, Transglutaminase-mediated oligomerization of the fibrin(ogen) αC domains promotes integrin-dependent cell adhesion and signaling. Blood 105, 3561–3568 (2005). 15637140 10.1182/blood-2004-10-4089 PMC1895018

[31] B. Li, C. Bechtler, L. Jenny, D. Ricklin, V. Schroeder, Exploring the function of factor XIII free B subunit: Interactions with complement factors and a novel approach to identify potential binding partners. Res. Pract. Thromb. Haemost. 6, e12766 (2022). 35873217 10.1002/rth2.12766 PMC9301527

[32] X. Tang, Z. Zhang, M. Fang, Y. Han, G. Wang, S. Wang, M. Xue, Y. Li, L. Zhang, J. Wu, B. Yang, J. Mwangi, Q. Lu, X. Du, R. Lai, Transferrin plays a central role in coagulation balance by interacting with clotting factors. Cell Res. 30, 119–132 (2020). 31811276 10.1038/s41422-019-0260-6 PMC7015052

[33] J. Koo, D. Galanakis, Y. Liu, A. Ramek, A. Fields, X. Ba, M. Simon, M. H. Rafailovich, Control of anti-thrombogenic properties: Surface-induced self-assembly of fibrinogen fibers. Biomacromolecules 13, 1259–1268 (2012). 22423652 10.1021/bm2015976

[34] P. R. Blanquet, Casein kinase 2 as a potentially important enzyme in the nervous system. Prog. Neurobiol. 60, 211–246 (2000). 10658642 10.1016/s0301-0082(99)00026-x

[35] S. Shiosaka, Kallikrein 8: A key sheddase to strengthen and stabilize neural plasticity. Neurosci. Biobehav. Rev. 140, 104774 (2022). 35820483 10.1016/j.neubiorev.2022.104774

[36] Y. C. Jang, R. Tsou, N. S. Gibran, F. F. Isik, Vitronectin deficiency is associated with increased wound fibrinolysis and decreased microvascular angiogenesis in mice. Surgery 127, 696–704 (2000). 10840366 10.1067/msy.2000.105858

[37] S. Chow, N. Di Girolamo, Vitronectin: A migration and wound healing factor for human corneal epithelial cells. Invest. Ophthalmol. Vis. Sci. 55, 6590–6600 (2014). 25237160 10.1167/iovs.14-15054

[38] X. Pang, X. He, Z. Qiu, H. Zhang, R. Xie, Z. Liu, Y. Gu, N. Zhao, Q. Xiang, Y. Cui, Targeting integrin pathways: Mechanisms and advances in therapy. Signal Transduct. Target Ther. 8, 1 (2023). 36588107 10.1038/s41392-022-01259-6 PMC9805914

[39] Y. Sun, S. Chatterjee, X. Lian, Z. Traylor, S. R. Sattiraju, Y. Xiao, S. A. Dilliard, Y. C. Sung, M. Kim, S. M. Lee, S. Moore, X. Wang, D. Zhang, S. Wu, P. Basak, J. Wang, J. Liu, R. J. Mann, D. F. LePage, W. Jiang, S. Abid, M. Hennig, A. Martinez, B. A. Wustman, D. J. Lockhart, R. Jain, R. A. Conlon, M. L. Drumm, C. A. Hodges, D. J. Siegwart, In vivo editing of lung stem cells for durable gene correction in mice. Science 384, 1196–1202 (2024). 38870301 10.1126/science.adk9428 PMC12208706

[40] R. K. Kainthan, M. Gnanamani, M. Ganguli, T. Ghosh, D. E. Brooks, S. Maiti, J. N. Kizhakkedathu, Blood compatibility of novel water soluble hyperbranched polyglycerol-based multivalent cationic polymers and their interaction with DNA. Biomaterials 27, 5377–5390 (2006). 16854460 10.1016/j.biomaterials.2006.06.021

[41] E. A. Jun, K. M. Lim, K. Kim, O. N. Bae, J. Y. Noh, K. H. Chung, J. H. Chung, Silver nanoparticles enhance thrombus formation through increased platelet aggregation and procoagulant activity. Nanotoxicology 5, 157–167 (2011). 20822370 10.3109/17435390.2010.506250

[42] E. Smyth, A. Solomon, A. Vydyanath, P. K. Luther, S. Pitchford, T. D. Tetley, M. Emerson, Induction and enhancement of platelet aggregation in vitro and in vivo by model polystyrene nanoparticles. Nanotoxicology 9, 356–364 (2015). 25030098 10.3109/17435390.2014.933902

[43] R. W. Farndale, J. J. Sixma, M. J. Barnes, P. G. de Groot, The role of collagen in thrombosis and hemostasis. J Thromb Haemost 2, 561–573 (2004). 15102010 10.1111/j.1538-7836.2004.00665.x

[44] J. Hirsh, S. S. Anand, J. L. Halperin, V. Fuster, Mechanism of action and pharmacology of unfractionated heparin. Arterioscler. Thromb. Vasc. Biol. 21, 1094–1096 (2001). 11451734 10.1161/hq0701.093686

[45] B. E. Berlioz, P. Patel, D. K. Sanghavi, “Bivalirudin” in *StatPearls* (StatPearls Publishing, 2025). 32491755

[46] A. N. Ilinskaya, M. A. Dobrovolskaia, Nanoparticles and the blood coagulation system. Part II: Safety concerns. Nanomedicine 8, 969–981 (2013). 23730696 10.2217/nnm.13.49 PMC3939602

[47] A. Vaidya, S. Moore, S. Chatterjee, E. Guerrero, M. Kim, L. Farbiak, S. A. Dilliard, D. J. Siegwart, Expanding RNAi to kidneys, lungs, and spleen via Selective ORgan Targeting (SORT) siRNA lipid nanoparticles. Adv. Mater. 36, e2313791 (2024). 38973655 10.1002/adma.202313791 PMC11823468

[48] P. K. Jadaun, S. Chatterjee, COVID-19 and dys-regulation of pulmonary endothelium: Implications for vascular remodeling. Cytokine Growth Factor Rev. 63, 69–77 (2022). 34728151 10.1016/j.cytogfr.2021.10.003 PMC9611904

[49] K. Suresh, L. A. Shimoda, Lung circulation. Compr. Physiol. 6, 897–943 (2016). 27065170 10.1002/cphy.c140049 PMC7432532

[50] Y. X. Li, H. B. Wang, J. Li, J. B. Jin, J. B. Hu, C. L. Yang, Targeting pulmonary vascular endothelial cells for the treatment of respiratory diseases. Front. Pharmacol. 13, 983816 (2022). 36110525 10.3389/fphar.2022.983816 PMC9468609

[51] P. G. Frank, S. E. Woodman, D. S. Park, M. P. Lisanti, Caveolin, caveolae, and endothelial cell function. Arterioscler. Thromb. Vasc. Biol. 23, 1161–1168 (2003). 12689915 10.1161/01.ATV.0000070546.16946.3A

[52] R. D. Minshall, W. C. Sessa, R. V. Stan, R. G. Anderson, A. B. Malik, Caveolin regulation of endothelial function. Am. J. Physiol. Lung Cell Mol. Physiol. 285, L1179–L1183 (2003). 14604847 10.1152/ajplung.00242.2003

[53] N. A. Maniatis, O. Chernaya, V. Shinin, R. D. Minshall, Caveolins and lung function. Adv. Exp. Med. Biol. 729, 157–179 (2012). 22411320 10.1007/978-1-4614-1222-9_11 PMC3449096

[54] J. H. Jones, E. Friedrich, Z. Hong, R. D. Minshall, A. B. Malik, PV1 in caveolae controls lung endothelial permeability. Am. J. Respir. Cell Mol. Biol. 63, 531–539 (2020). 32663411 10.1165/rcmb.2020-0102OC PMC7528930

[55] J. Voigt, J. Christensen, V. P. Shastri, Differential uptake of nanoparticles by endothelial cells through polyelectrolytes with affinity for caveolae. Proc. Natl. Acad. Sci. U.S.A. 111, 2942–2947 (2014). 24516167 10.1073/pnas.1322356111 PMC3939899

[56] H. Shu, Y. Peng, W. Hang, J. Nie, N. Zhou, D. W. Wang, The role of CD36 in cardiovascular disease. Cardiovasc. Res. 118, 115–129 (2022). 33210138 10.1093/cvr/cvaa319 PMC8752351

[57] I. Gracia-Rubio, C. Martin, F. Civeira, A. Cenarro, SR-B1, a key receptor involved in the progression of cardiovascular disease: A perspective from mice and human genetic studies. Biomedicines 9, 612 (2021). 34072125 10.3390/biomedicines9060612 PMC8229968

[58] D. K. Strickland, D. T. Au, P. Cunfer, S. C. Muratoglu, Low-density lipoprotein receptor-related protein-1: Role in the regulation of vascular integrity. Arterioscler. Thromb. Vasc. Biol. 34, 487–498 (2014). 24504736 10.1161/ATVBAHA.113.301924 PMC4304649

[59] R. Ma, R. Xie, C. Yu, Y. Si, X. Wu, L. Zhao, Z. Yao, S. Fang, H. Chen, V. Novakovic, C. Gao, J. Kou, Y. Bi, H. S. Thatte, B. Yu, S. Yang, J. Zhou, J. Shi, Phosphatidylserine-mediated platelet clearance by endothelium decreases platelet aggregates and procoagulant activity in sepsis. Sci. Rep. 7, 4978 (2017). 28694452 10.1038/s41598-017-04773-8 PMC5504060

[60] K. T. Love, K. P. Mahon, C. G. Levins, K. A. Whitehead, W. Querbes, J. R. Dorkin, J. Qin, W. Cantley, L. L. Qin, T. Racie, M. Frank-Kamenetsky, K. N. Yip, R. Alvarez, D. W. Sah, A. de Fougerolles, K. Fitzgerald, V. Koteliansky, A. Akinc, R. Langer, D. G. Anderson, Lipid-like materials for low-dose, in vivo gene silencing. Proc. Natl. Acad. Sci. U.S.A. 107, 1864–1869 (2010). 20080679 10.1073/pnas.0910603106 PMC2804742

[61] M. Jayaraman, S. M. Ansell, B. L. Mui, Y. K. Tam, J. Chen, X. Du, D. Butler, L. Eltepu, S. Matsuda, J. K. Narayanannair, K. G. Rajeev, I. M. Hafez, A. Akinc, M. A. Maier, M. A. Tracy, P. R. Cullis, T. D. Madden, M. Manoharan, M. J. Hope, Maximizing the potency of siRNA lipid nanoparticles for hepatic gene silencing in vivo. Angew. Chem. Int. Ed. Engl. 51, 8529–8533 (2012). 22782619 10.1002/anie.201203263 PMC3470698

[62] O. M. Al-Amer, The role of thrombin in haemostasis. Blood Coagul. Fibrinolysis 33, 145–148 (2022). 35239615 10.1097/MBC.0000000000001130

[63] S. Ilveskero, P. Siljander, R. Lassila, Procoagulant activity on platelets adhered to collagen or plasma clot. Arterioscler. Thromb. Vasc. Biol. 21, 628–635 (2001). 11304482 10.1161/01.atv.21.4.628

[64] J. Winkler, Oligonucleotide conjugates for therapeutic applications. Ther. Deliv. 4, 791–809 (2013). 23883124 10.4155/tde.13.47 PMC3787477

[65] K. Neubauer, B. Zieger, Endothelial cells and coagulation. Cell Tissue Res. 387, 391–398 (2022). 34014399 10.1007/s00441-021-03471-2 PMC8975780

[66] Q. Li, C. Chan, N. Peterson, R. N. Hanna, A. Alfaro, K. L. Allen, H. Wu, W. F. Dall’Acqua, M. J. Borrok, J. L. Santos, Engineering caveolae-targeted lipid nanoparticles to deliver mRNA to the lungs. ACS Chem. Biol. 15, 830–836 (2020). 32155049 10.1021/acschembio.0c00003

[67] A. B. Banizs, T. Huang, K. Dryden, S. S. Berr, J. R. Stone, R. K. Nakamoto, W. Shi, J. He, In vitro evaluation of endothelial exosomes as carriers for small interfering ribonucleic acid delivery. Int. J. Nanomedicine 9, 4223–4230 (2014). 25214786 10.2147/IJN.S64267 PMC4159392

[68] K. O’Brien, K. Breyne, S. Ughetto, L. C. Laurent, X. O. Breakefield, RNA delivery by extracellular vesicles in mammalian cells and its applications. Nat. Rev. Mol. Cell Biol. 21, 585–606 (2020). 32457507 10.1038/s41580-020-0251-y PMC7249041

[69] D. Chen, K. T. Love, Y. Chen, A. A. Eltoukhy, C. Kastrup, G. Sahay, A. Jeon, Y. Dong, K. A. Whitehead, D. G. Anderson, Rapid discovery of potent siRNA-containing lipid nanoparticles enabled by controlled microfluidic formulation. J. Am. Chem. Soc. 134, 6948–6951 (2012). 22475086 10.1021/ja301621z

