# Supplementary material for: Activation of the coagulation cascade as a mechanism for selective nanoparticle-mediated RNA delivery to the endothelium in vivo
Source: Sci Adv. 2025 Oct 22;11(43):eady2738. doi: 10.1126/sciadv.ady2738 (PMC12542961; doi:10.1126/sciadv.ady2738)
Supplement: Supplementary file 1 — Figs. S1 to S8 Tables S1 to S5 [file sciadv.ady2738_sm.pdf]

Science Advances
AAAS

Supplementary Materials for

Activation of the coagulation cascade as a mechanism for selective nanoparticle-mediated RNA delivery to the endothelium in vivo

Edward B. Guzman *et al.*

Corresponding author: Daniel G. Anderson, dgander@mit.edu

Sci. Adv. **11**, eady2738 (2025)
DOI: 10.1126/sciadv.ady2738

This PDF file includes:

Figs. S1 to S8
Tables S1 to S5

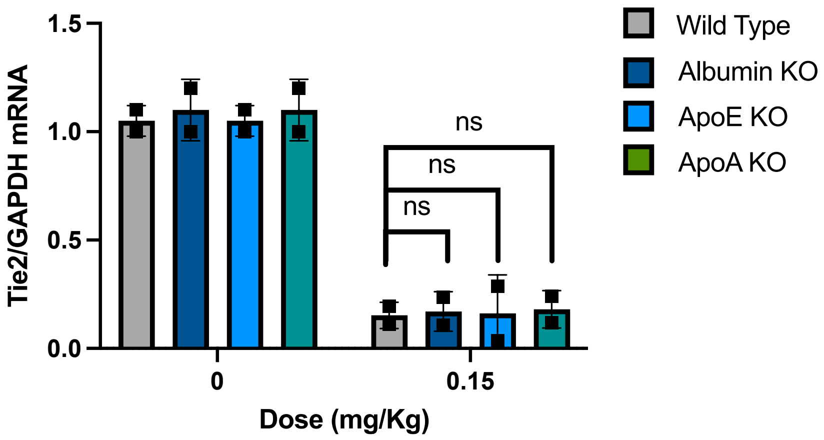

Supplementary Figure 1: Non-essential proteins for pulmonary RNA delivery by nanoparticles preferential to endothelial cells. Tie2 knockdown in the lungs of Albumin, ApoE, or ApoA knockout (KO) and wildtype mice by NPE-1 nanoparticles 48hrs after NP administration into the tail vein. Nanoparticles were formulated with siTie2 and injected at 0.15 mg/kg of siRNA. Data is shown as mean +/- s.d. N= 2 per group. n.s. = not statistically significant when $P > 0.05$ using a two-tailed, unpaired t-test. Biological replicates shown.

A.

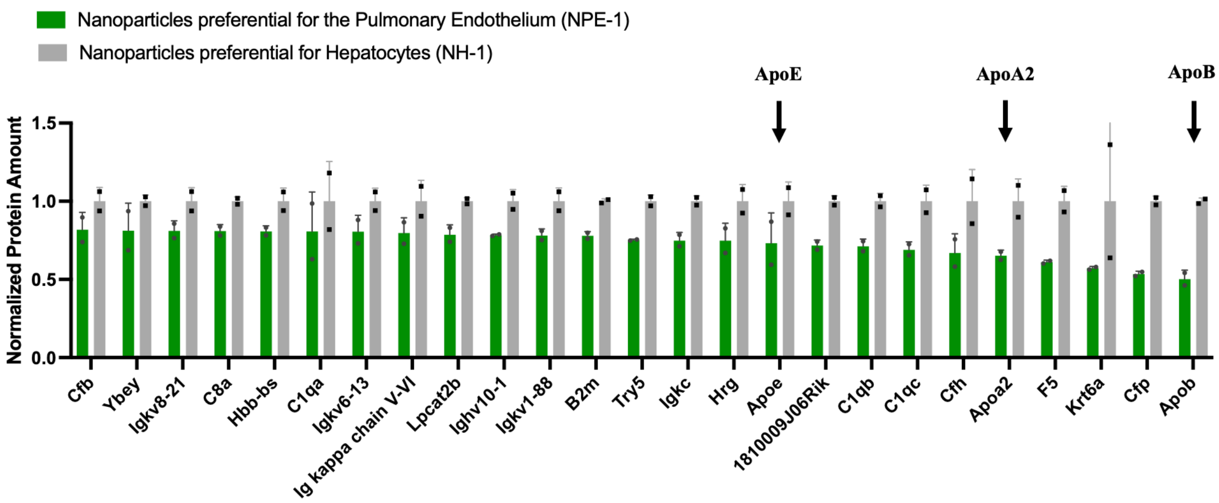

B.

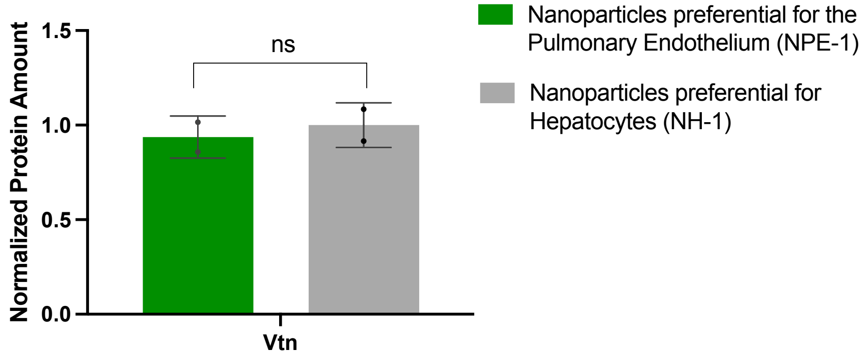

Supplementary Figure 2: Least preferential proteins for endothelial nanoparticles. (A) Normalized protein abundance from the corona of NPE-1 nanoparticles relative to the corona proteins from NH-1 nanoparticles, demonstrating that apoE and other apolipoproteins have the lowest affinity to endothelial nanoparticles. The least 25 preferential proteins to NPE-1 nanoparticles are shown. (B) Abundance of vitronectin (Vtn) in the corona of NPE-1 nanoparticles relative to the abundance of vitronectin from the corona of NH-1 nanoparticles. Data is shown as mean +/- s.d, and n.s. = not statistically significant when $P > 0.05$ using a two-tailed, unpaired t-test.

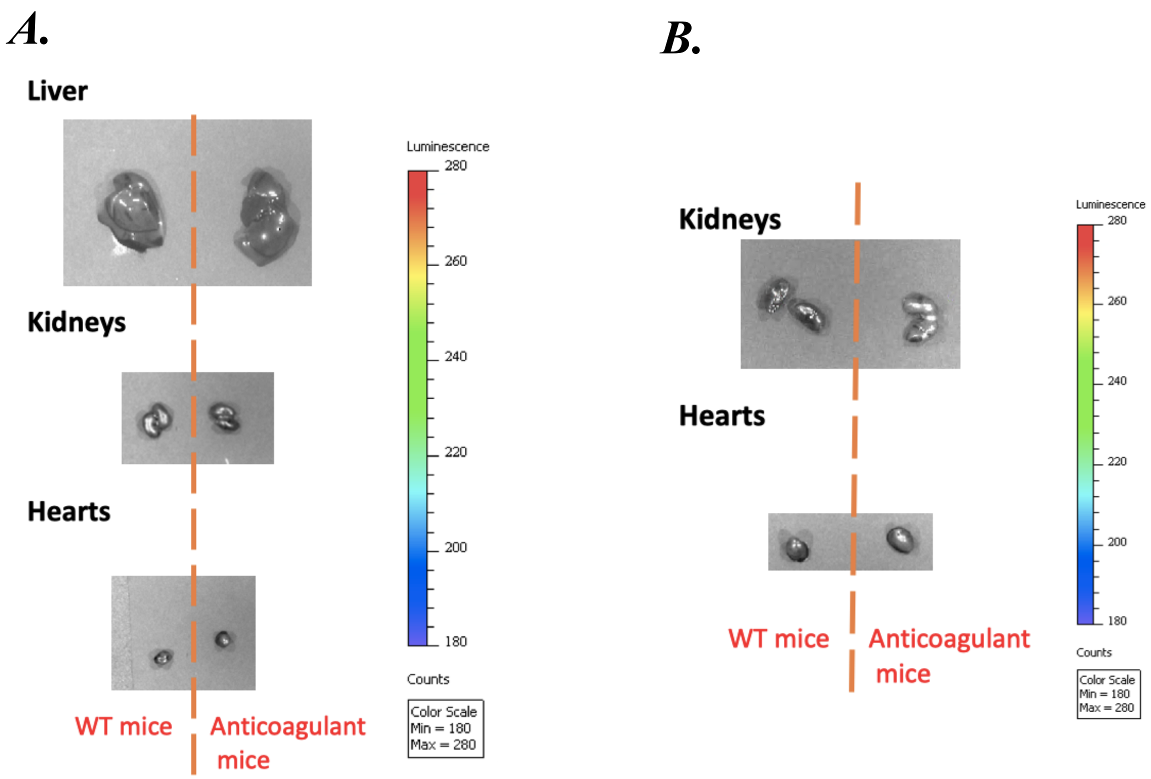

Supplementary Figure 3: Nanoparticles are not redirected from lung to other organs in heparin treated mice. (A) No luciferase expression is observed in the liver, kidneys, or heart of WT mice or anticoagulant mice 6 hrs after injection of NPE-3 nanoparticles encapsulating Luciferase mRNA. Luciferase expression was assessed based on luminesce radiance. (B) No luciferase expression is observed in the kidneys or heart of anticoagulant mice or wild type mice 6 hrs after injection of NPE-4 nanoparticles encapsulating Luciferase mRNA. N = 2-3 mice per group. Biological replicate shown.

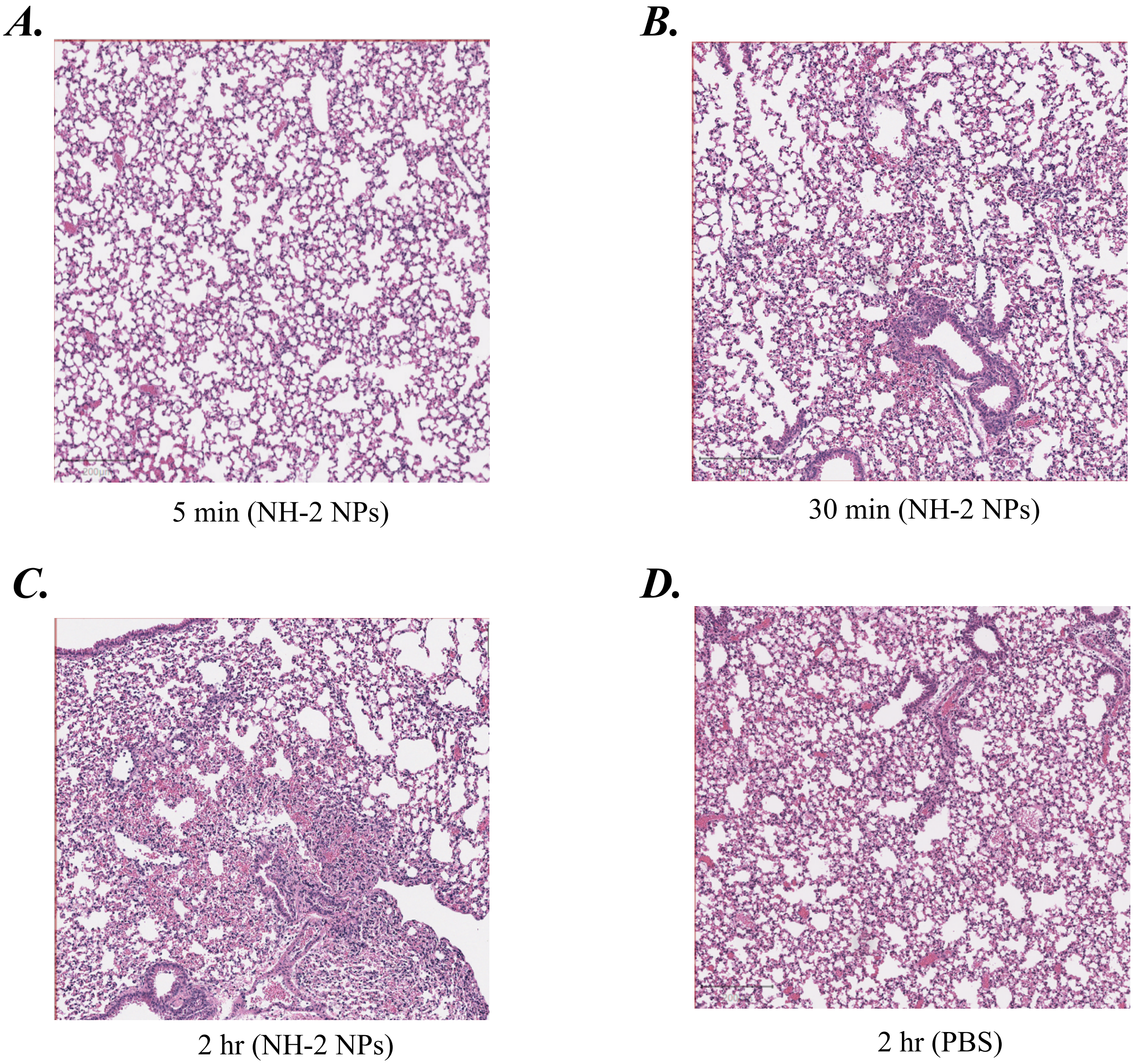

Supplementary Figure 4: Representative posttreatment histology images from wildtype mice treated with nanoparticles preferential to hepatocytes or with PBS. Hematoxylin and eosin were used to stain the tissue. No signs of vessel occlusion or clots were observed during a 48 hr period after nanoparticle administration. (A-C) Lung from mice treated with NH-2 nanoparticles at the indicated time points post administration. Nanoparticles were administered at 1 mg/kg of RNA intravenously into the tail vein. (D) Lung from mice treated with PBS at 2 hrs after a tail vein administration. N=2 mice per group. Biological replicate shown.

A. *B.*

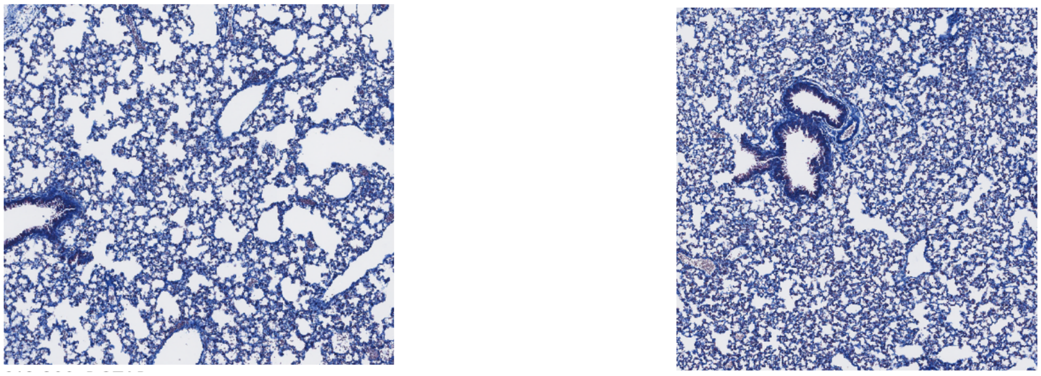

2 hr (NPE-5 NPs) 2 hr (NH-2 NPs)

Supplementary Figure 5: Representative posttreatment histology images from wildtype mice treated with nanoparticles preferential to endothelial cells or hepatocytes. Carstair staining was used to stain the tissue (platelets: gray; fibrin: orange; collagen: blue; red blood cells and muscle: red). No signs of vessel occlusion or clots were observed after nanoparticle administration. (A) Lung from mice treated with NPE-5 at the indicated time point post IV administration. (B) Lung from mice treated with NH-2 at the indicated time point after a tail vein administration. Nanoparticles were administered at 1 mg/kg of RNA intravenously into the tail vein. N=2 mice per group. Biological replicate shown.

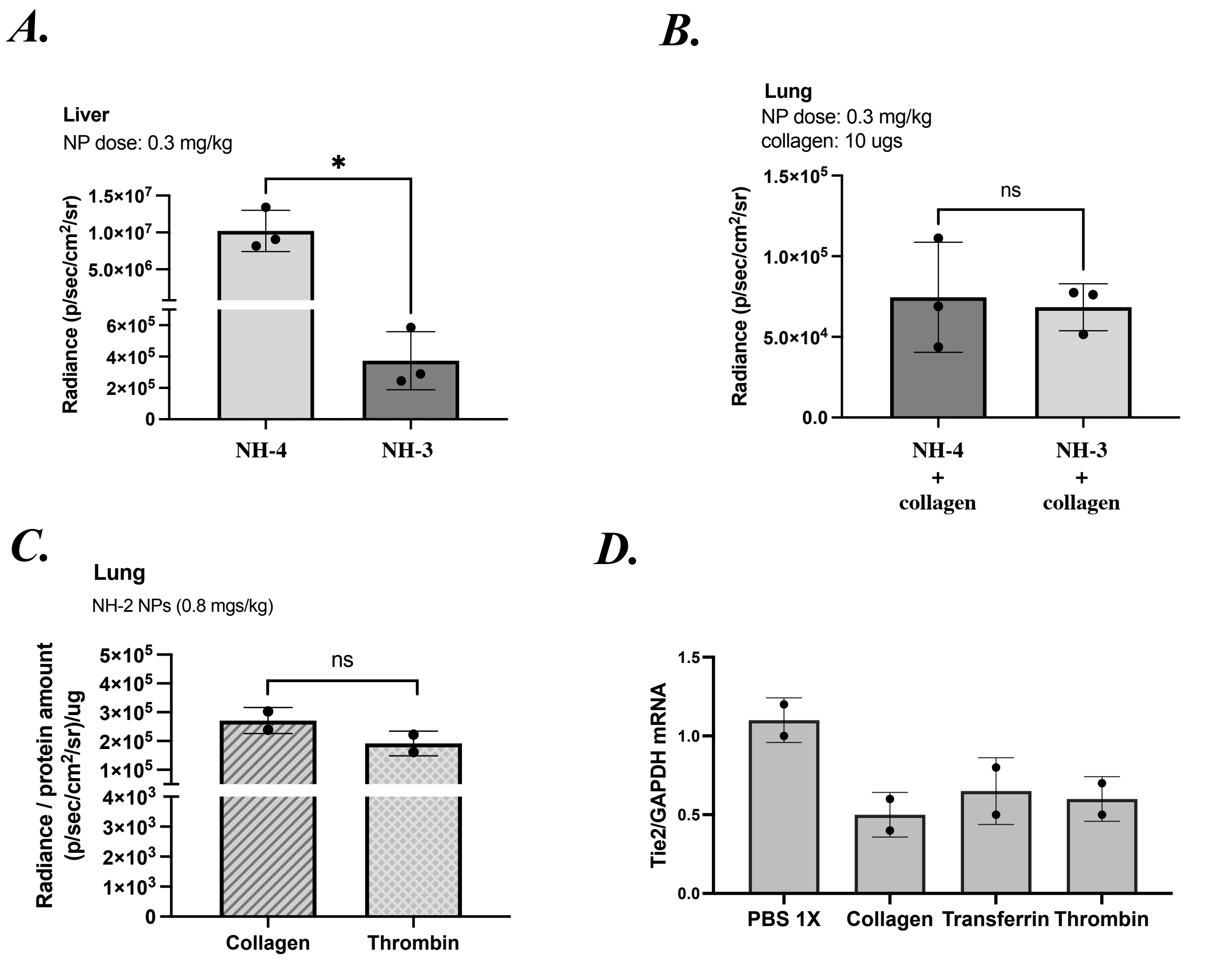

Supplementary Figure 6: Procoagulant protein and lipid comparison for pulmonary transfection. (A) NH-3 and NH-4 nanoparticles were formulated with luciferase mRNA and the ionizable lipid MC3 or cKK-E12, respectively, for i.v administration. Liver transfection measured in the form of luminescent radiance from the liver was higher for NH-4 than NH-3 nanoparticles. NPs were injected at 0.3 mg/kg of mRNA and luciferase expression was evaluated 6 hours after IV injection. (B) NH-3 or NH-4 nanoparticles containing the ionizable lipid MC3 or cKK-E12, respectively, were incubated with 10 ugs of collagen for 30 min prior to i.v. administration for pulmonary RNA delivery. Nanoparticles were injected at 0.3 mg/kg of luciferase mRNA with 10 ugs of collagen. Luciferase expression in the lung was evaluated 6 hours after IV injection, demonstrating that both nanoparticles produce equivalent transfection levels. (C) NH-2 nanoparticles were formulated with luciferase mRNA and pre-incubated for 30 min with 10 ugs of collagen or 1 ug of thrombin prior to IV injection at 0.8 mg/kg of mRNA. Luciferase expression in the lung was evaluated 6 hours after IV injection. (D) Tie2 expression after treatment with NH-4 nanoparticles formulated with siTie2 and pre-incubated with pro-coagulant collagen (10ugs), transferrin (50ugs), or thrombin (1 ugs). Nanoparticles were injected intravenously, and organs were collected 48 hrs after administration. Data is shown as mean +/- s.d. N = 2-3 per group. $^*P < 0.05$ and n.s. = not statistically significant when $P > 0.05$ using a two-tailed, unpaired t-test. Biological replicates shown.

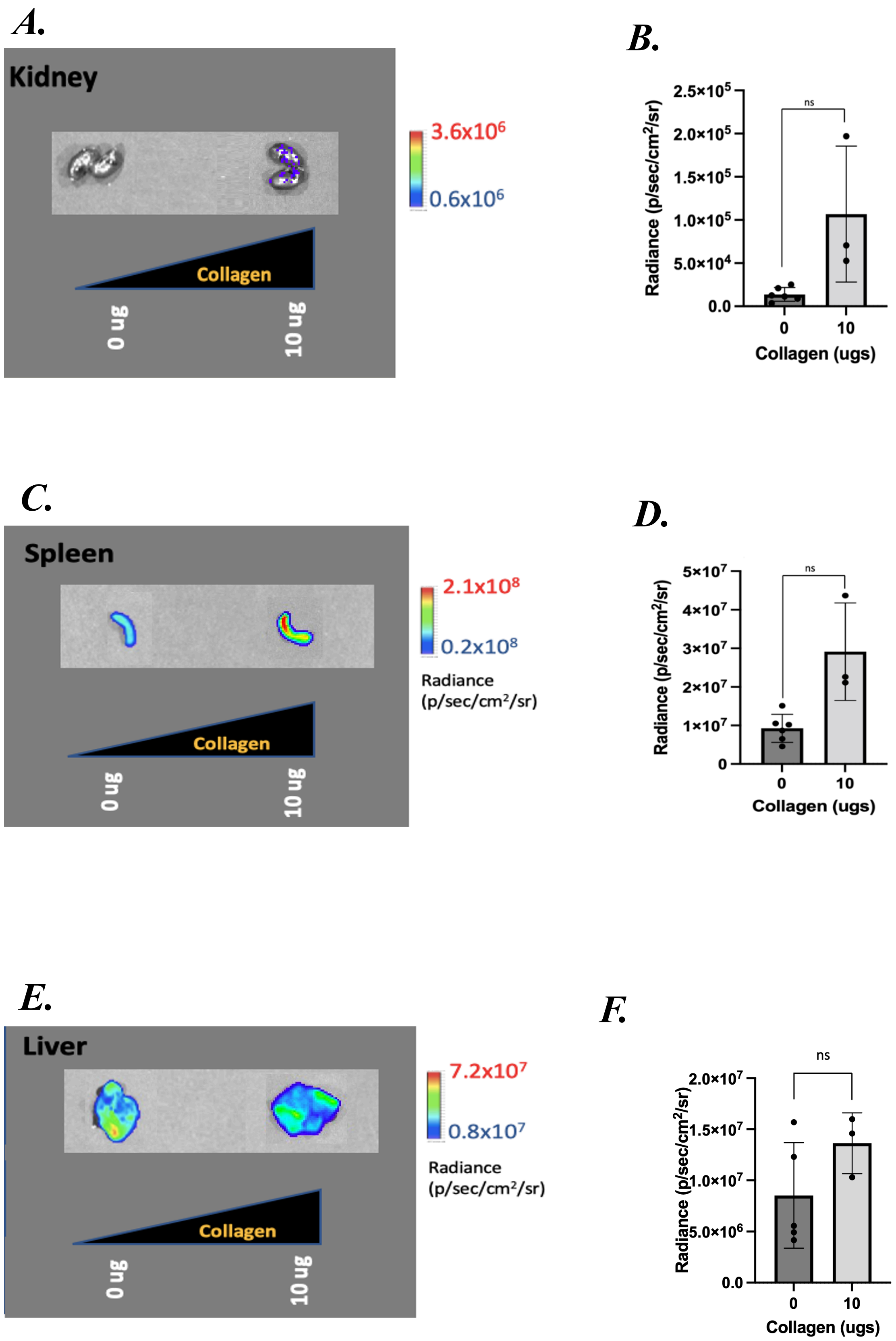

A.
Kidney
3.6x10^6
0.6x10^6
Collagen
0 ug
10 ug
B.
Radiance (p/sec/cm²/sr)
2.5×10^5
2.0×10^5
1.5×10^5
1.0×10^5
5.0×10^4
0.0
ns
0
10
Collagen (ugs)
C.
Spleen
2.1x10^8
0.2x10^8
Radiance
(p/sec/cm²/sr)
Collagen
0 ug
10 ug
D.
Radiance (p/sec/cm²/sr)
5×10^7
4×10^7
3×10^7
2×10^7
1×10^7
0
ns
0
10
Collagen (ugs)
E.
Liver
7.2x10^7
0.8x10^7
Radiance
(p/sec/cm²/sr)
Collagen
0 ug
10 ug
F.
Radiance (p/sec/cm²/sr)
2.0×10^7
1.5×10^7
1.0×10^7
5.0×10^6
0.0
ns
0
10
Collagen (ugs)

Supplementary Figure 7: Transfection of different organs by NH-2 nanoparticles pre-incubated with procoagulant collagen. (A) Luciferase expression in the kidneys by NH-2 nanoparticles formulated with luciferase mRNA and pre-incubated for 30 min with collagen prior to IV injection. NPs were injected at 0.8 mg/kg of mRNA with 0 or 10 ugs of collagen and transfection was evaluated 6 hours after IV injection. (B) Luminescence quantification in the kidneys of mice treated with NH-2 nanoparticles pre-incubated with collagen. Nanoparticles were injected at 0.8 mg/kg of mRNA with 0 or 10 ugs of collagen. (C) Luciferase expression in the spleen by NH-2 nanoparticles formulated with luciferase mRNA and pre-incubated for 30 min with collagen prior to IV injection. NPs were injected at 0.8 mg/kg of mRNA with 0 or 10 ugs of collagen and transfection was evaluated 6 hours after IV injection. (D) Luminescence quantification in the spleen of mice treated with NH-2 nanoparticles pre-incubated with collagen. Nanoparticles were injected at 0.8 mg/kg of mRNA with 0 or 10 ugs of collagen. (E) Luciferase expression in the liver by NH-2 nanoparticles formulated with luciferase mRNA and pre-incubated for 30 min with collagen prior to IV injection. NPs were injected at 0.8 mg/kg of mRNA with 0 or 10 ugs of collagen and transfection was evaluated 6 hours after IV injection. (F) Luminescence quantification in the liver of mice treated with NH-2 nanoparticles pre-incubated with collagen. Nanoparticles were injected at 0.8 mg/kg of mRNA with 0 or 10 ugs of collagen. Data is shown as mean +/- s.d. n.s. = not statistically significant when $P > 0.05$ using a two-tailed, unpaired t-test. N=3-6 mice per group. Biological replicate shown.

A.

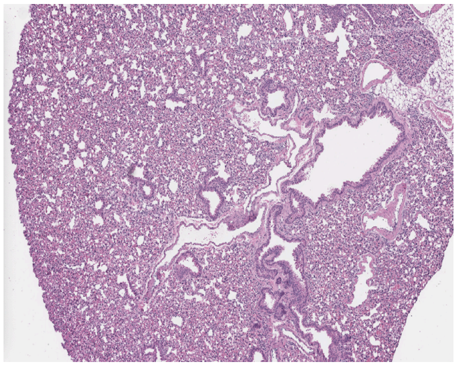

24 hrs (NH-2 + collagen)

B.

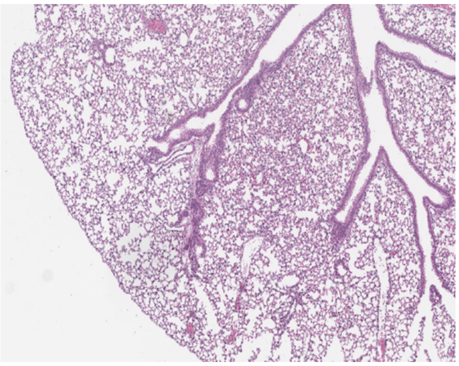

24 hrs (PBS 1X)

Supplementary Figure 8: Representative posttreatment histology images from wildtype mice treated with NH nanoparticles pre-incubated with procoagulant proteins or with PBS. Hematoxylin and eosin were used to stain the tissue. Signs of vessel occlusion and inflammation were observed after nanoparticle administration. (A) Lung from mice treated with NH-2 nanoparticles pre-incubated with collagen at 24 hrs post administration. Nanoparticles were administered at 0.8 mg/kg of mRNA with 10 ugs of collagen intravenously into the tail vein. (B) Lung from mice treated with PBS at 24 hrs after a tail vein administration. N=2 mice per group. Biological replicate shown.

Supplementary Table 1: Nanoparticles Physicochemical Properties

Nanoparticle	Size (nm)	PDI	Z-potential, ζ (mV)
NPE-1	110	0.15	-3
NPE-2	60	0.05	12
NPE-3	90	0.11	-1
NPE-4	63	0.06	2
NPE-5	68	0.08	5
NH-1	65	0.05	-1
NH-2	63	0.06	-2
NH-3	68	0.07	-2
NH-4	67	0.05	-1

Supplementary Table 2: Nanoparticle size after incubation with platelet-poor plasma

Nanoparticle	Size (nm)	PDI
NPE-1 + plasma	130	0.25
NPE-5 + plasma	108	0.18
NH-2 + plasma	84	0.16

Supplementary Table 3: Nanoparticle size after pre-incubation with pro-coagulant proteins

Nanoparticle	Size (nm)	PDI
NH-2 + collagen	69	0.18
NH-3 + collagen	73	0.14
NH-4 + collagen	70	0.16
NH-2 + transferrin	71	0.18
NH-2 + thrombin	70	0.16

Supplementary Table 4 : Absolute protein amount from each protein identified on the corona of nanoparticles preferential to the pulmonary endothelium (NPE-1)

	Accession	Description	Gene	Intensity	STD
1	P07724	Albumin GN=Alb	Alb	9857247357	626717543
2	Q921I1	Serotransferrin GN=Tf	Tf	1183647124	18139073
3	Q8K0E8	Fibrinogen beta chain GN	Fgb	903437807	392100852
4	E9PV24	Fibrinogen alpha chain G	Fga	870087923	375534602
5	Q61838	Pregnancy zone protein G	Pzp	655362297	183021912
6	Q00623	Apolipoprotein A-I GN=A	Apoa1	574025544	471281891
7	P01027	Complement C3 GN=C3	C3	515527273	48747749
8	Q00897	Alpha-1-antitrypsin 1-4 G	Serpina1d	446432534	57391181
9	Q8VCM7	Fibrinogen gamma chain	Fgg	391565972	257528837
10	A0A0R4J0I1	Serine protease inhibitor	Serpina3k	292771890	20675973
11	P28665	Murinoglobulin-1 GN=M	Mug1	259465120	64502245
12	P06728	Apolipoprotein A-IV GN=	Apoa4	231031635	162539487
13	P23953	Carboxylesterase 1C GN=	Ces1c	218785802	7671756
14	P19221	Prothrombin GN=F2	F2	208629118	62264981
15	A0A075B5P6	Immunoglobulin heavy c	Ighm	182989939	14039430
16	P06909	Complement factor H GN	Cfh	168168555	50672539
17	O08677	Kininogen-1 GN=Kng1	Kng1	139837702	21596888
18	P11276	Fibronectin GN=Fn1	Fn1	123565145	16158758
19	G3X8Q5	Ceruloplasmin GN=Cp	Cp	120892709	18963348
20	P29699	Alpha-2-HS-glycoprotein	Ahsg	120538828	4045423
21	P08226	Apolipoprotein E GN=Ap	Apoe	119809444	51662640
22	Q91X72	Hemopexin GN=Hpx	Hpx	118129792	17024657
23	P20918	Plasminogen GN=Plg	Plg	98739885	7615460
24	Q06890	Clusterin GN=Clu	Clu	98562816	67160370
25	B7ZCR0	Uridine-cytidine kinase-l	Uckl1	96972257	10464442
26	P13020	Gelsolin GN=Gsn	Gsn	91052557	17628201
27	P01029	Complement C4-B GN=C4	C4b	75064732	27539204
28	O89020	Afamin GN=Afm	Afm	72939771	7469232
29	P07309	Transthyretin GN=Ttr	Ttr	67754273	14904286
30	Q61703	Inter-alpha-trypsin inhib	Itih2	64826161	19122295
31	P01837	Immunoglobulin kappa c	Igkc	61406294	6985585
32	A0A0R4J0X5	Alpha-1-antitrypsin 1-3 G	Serpina1c	59671519	17393232
33	P97290	Plasma protease C1 inhib	Serping1	58971475	7520597
34	P21614	Vitamin D-binding prote	Gc	57488771	26762322
35	F8WJ05	Inter-alpha-trypsin inhib	Itih1	53586700	16910528
36	P03953	Complement factor D GN	Cfd	51204686	51866743
37	E9Q5L2	Inter alpha-trypsin inhib	Itih4	50245441	4808800
38	P32261	Antithrombin-III GN=Ser	Serpinc1	48609693	417464
39	A8DUK4	Beta-globin GN=Hbb-bs	Hbb-bs	47638506	3436077
40	P06684	Complement C5 GN=C5	C5	43685770	17015965
41	Q07456	Protein AMBP GN=Ambp	Ambp	35165670	6215359
42	Q9QUK9	TESP4 GN=Try5	Try5	31682657	348496
43	A0A2I3BRQ3	Inter-alpha-trypsin inhib	Itih3	31083029	8397669
44	P04186	Complement factor B GN	Cfb	28418777	6289002
45	A0A0A6YVP0	Immunoglobulin heavy c	Ighg2b	27579102	1586228

46	P52430	Serum paraoxonase/aryl	Pon1	25691855	10987885
47	Q61247	Alpha-2-antiplasmin GN=	Serpinf2	24280941	2443235
48	P29788	Vitronectin GN=Vtn	Vtn	23793570	4598258
49	Q61129	Complement factor I GN	Cfi	22842252	2705059
50	E9QP56	Apolipoprotein C-III GN=	Apoc3	22628052	6986825
51	Q02257	Junction plakoglobin GN	Jup	21632073	22892543
52	Q9DBD0	Inhibitor of carbonic anh	Ica	20787742	1691840
53	P09813	Apolipoprotein A-II GN=A	Apoa2	19846387	1785037
54	Q91VB8	Alpha globin 1 GN=Hba-a	Hba-a1	19542431	785885
55	Q01339	Beta-2-glycoprotein 1 GN	Apoh	19339346	798177
56	Q60590	Alpha-1-acid glycoprotei	Orm1	18547526	12115641
57	A0A0A6YY53	Immunoglobulin heavy c	Ighg2c	18155162	7369728
58	P22599	Alpha-1-antitrypsin 1-2 G	Serpina1b	17359088	54615
59	A0A338P699	Kininogen 2 (Fragment) G	Kng2	16366653	927391
60	P01898	H-2 class I histocompatib	H2-Q10	15769642	6191893
61	Q9Z1R3	Apolipoprotein M GN=Ap	Apom	14480279	1683478
62	A0A1Y7VJN6	Immunoglobulin heavy c	Ighg3	14288207	3368467
63	A0A0A6YWR2	Ig gamma-1 chain C regio	Ighg1	14195732	1779631
64	P41317	Mannose-binding protei	Mbl2	14057065	3950064
65	P34928	Apolipoprotein C-I GN=A	Apoc1	13259087	4268940
66	A0A0R4J039	Histidine-rich glycoprote	Hrg	12779626	3100448
67	P26262	Plasma kallikrein GN=Klk	Klkb1	11967718	1972469
68	Q19LI2	Alpha-1B-glycoprotein G	A1bg	11875359	940094
69	Q80YC5	Coagulation factor XII GN	F12	11464679	3813324
70	P01646	Ig kappa chain V-V region	0	11462844	1785160
71	Q8VCU2	Glycosyl-phosphatidylin	Gpld1	10692960	1727057
72	A0A075B5V1	Immunoglobulin heavy v	Ighv1-31	10218958	1610330
73	Q9DBB9	Carboxypeptidase N sub	Cpn2	9690583	405048
74	P46412	Glutathione peroxidase 3	Gpx3	9653787	1795452
75	P42703	Leukemia inhibitory fact	Lifr	9641625	1531079
76	O88947	Coagulation factor X GN=	F10	8696176	1275863
77	Q6YJU1	Fetuin-B GN=Fetub	Fetub	8695946	425058
78	P01723	Ig lambda-1 chain V regio	0	8454442	937653
79	P14847	C-reactive protein GN=Cr	Crp	7860368	1757259
80	Q64726	Zinc-alpha-2-glycoprotei	Azgp1	7621983	1754443
81	E9Q1Y3	Apolipoprotein B-100 (F	Apob	7450388	1374851
82	P49182	Heparin cofactor 2 GN=S	Serpind1	7360649	2443079
83	Q06770	Corticosteroid-binding g	Serpina6	6637975	296814
84	A0A075B5N7	Immunoglobulin kappa v	Igkv6-13	6403457	1376427
85	Q9QWK4	CD5 antigen-like GN=Cd5	Cd5l	5664192	2318858
86	Q01279	Epidermal growth factor	Egfr	5621287	1044924
87	O70165	Ficolin-1 GN=Fcn1	Fcn1	5534588	1358121
88	A0A0A6YXW6	Immunoglobulin heavy c	Igha	5440645	135302
89	A0A140T8P7	Immunoglobulin kappa v	Igkv8-21	5334872	693829
90	P04940	Ig kappa chain V-VI regio	0	5249846	1632282
91	E9Q6I2	Interleukin-1 receptor ac	Il1rap	5175290	396925
92	P39039	Mannose-binding protei	Mbl1	4991619	622744
93	P60710	Actin, cytoplasmic 1 GN=	Actb	4797530	2804062

94	P11680	Properdin GN=Cfp	Cfp	4727129	266489
95	Q8BY71	Histone acetyltransferase	Hat1	4335515	6354237
96	P01679	Ig kappa chain V-VI regio	0	4203937	836891
97	P51885	Lumican GN=Lum	Lum	3820101	1076666
98	Q60994	Adiponectin GN=Adipoq	Adipoq	3786262	1611349
99	P70274	Selenoprotein P GN=Sele	Selenop	3716303	1016229
100	Q8BH61	Coagulation factor XIII A	F13a1	3489265	376136
101	Q9D7S9	Charged multivesicular b	Chmp5	3475447	107699
102	Q8CG14	Complement C1s-A subco	C1sa	3395215	1059388
103	P31532	Serum amyloid A-4 prote	Saa4	3247663	684692
104	A0A0G2JEU7	Immunoglobulin heavy v	Ighv1-82	3160353	1182557
105	A2A998	Complement componen	C8a	2976092	245529
106	A0A2R8VHR3	Mannan-binding lectin se	Masp1	2937116	332765
107	F8WI14	Extracellular matrix prot	Ecm1	2893426	655308
108	Q9JJN5	Carboxypeptidase N cata	Cpn1	2750923	462970
109	Q8CJ96	Ras association domain-c	Rassf8	2699748	8075
110	Q9Z1T2	Thrombospondin-4 GN=T	Thbs4	2690964	878892
111	Q08879	Fibulin-1 GN=Fbln1	Fbln1	2671719	297468
112	Q8R121	Protein Z-dependent pro	Serpina10	2500297	525085
113	P01657	Ig kappa chain V-III regio	0	2428232	944437
114	A0A075B5Y4	Immunoglobulin heavy v	Ighv1-81	2365800	494067
115	P51910	Apolipoprotein D GN=Ap	Apod	2244835	1183053
116	G3X8T9	Serine protease inhibitor	Serpina3n	2192534	78378
117	Q91XL1	Leucine-rich HEV glycopr	Lrg1	2166676	34843
118	Q5SX59	2-phospho-D-glycerate h	Eno3	2127310	1517414
119	Q4FZE8	Major urinary protein 1 G	Mup22	2118533	1055607
120	A0A140T8N3	Immunoglobulin kappa c	Igkv13-84	2113763	760224
121	Q07968	Coagulation factor XIII B	F13b	1961631	591710
122	A0A1B0GSK1	Ufm1-specific protease 2	Ufsp2	1912550	415036
123	O35608	Angiopoietin-2 GN=Angp	Angpt2	1909867	944372
124	Q9CPN7	RIKEN cDNA 1810009J06	810009J06Ri	1906885	155701
125	P50446	Keratin, type II cytoskele	Krt6a	1874634	59767
126	Q00724	Retinol-binding protein 4	Rbp4	1779905	234705
127	Q02105	Complement C1q subcon	C1qc	1731707	208044
128	P01887	Beta-2-microglobulin GN	B2m	1687467	93960
129	P08607	C4b-binding protein GN=	C4bpa	1657568	528913
130	Q8BYZ7	Engulfment and cell mot	Elmo3	1643608	26069
131	A0A2I3BRS7	Krueppel-like factor 10 G	Klf10	1643261	694174
132	E9Q5F6	Polyubiquitin-C (Fragme	Ubc	1597253	1084751
133	A0A1D5RLD8	Glyceraldehyde-3-phosp	Gm10358	1569989	1134213
134	P04104	Keratin, type II cytoskele	Krt1	1460264	1359292
135	P14106	Complement C1q subcon	C1qb	1449734	149577
136	P16301	Phosphatidylcholine-ste	Lcat	1449431	345231
137	P01665	Ig kappa chain V-III regio	0	1341830	741648
138	A0A140T8M2	Immunoglobulin kappa v	Igkv12-44	1336055	507770
139	Q9DAC2	Complement componen	C8g	1334336	298704
140	Q8BND5	Sulfhydryl oxidase 1 GN=	Qsox1	1318864	278475
141	Q05020	Apolipoprotein C-II GN=A	Apoc2	1292631	683334

142	A0A140T8Q3	Immunoglobulin kappa v	Igkv6-17	1284359	255282
143	Q8CG16	Complement C1r-A subc	C1ra	1282804	364923
144	A0A140T8P5	Immunoglobulin kappa c	Igkv8-24	1203309	173182
145	A0A0R4J0S2	Insulin-like growth facto	Igfals	1142493	645984
146	P06683	Complement componen	C9	1142215	146474
147	A0A140T8N1	Immunoglobulin kappa c	Igkv1-88	1123739	98797
148	P12246	Serum amyloid P-compo	Apcs	1044550	50927
149	A0A075B5N9	Immunoglobulin kappa v	Igkv3-7	983089	458879
150	Q08761	Vitamin K-dependent pr	Pros1	974708	249582
151	Q9CPN9	RIKEN cDNA 2210010C0	210010C04Ri	973230	298411
152	P01592	Immunoglobulin J chain	Jchain	912793	160127
153	P98086	Complement C1q subcor	C1qa	877391	446249
154	A0A3Q4EC30	Peptidase inhibitor 16 (F	Pi16	865139	310669
155	P70663	SPARC-like protein 1 GN=	Sparcl1	781754	252222
156	A0A075B677	Immunoglobulin kappa v	Igkv4-53	694887	473876
157	A0A571BF69	Maltase-glucoamylase GI	Mgam	688388	301229
158	A0A0G2JFA8	Immunoglobulin kappa v	Igkv17-121	682229	290157
159	E9PUM5	Predicted gene 4788 GN=	Gm4788	666794	114103
160	Q9D5U0	Lysophosphatidylcholin	Lpcat2b	651044	85820
161	A0A0B4J1J6	Immunoglobulin heavy v	Ighv10-1	638812	9104
162	Q792Z1	Trypsin 10 GN=Try10	Try10	606289	184117
163	E0CZ49	Mitochondrial tRNA-spe	Trmu	596764	308473
164	S4R2K3	Protein-L-isoaspartate O	Pcmtd1	588007	301270
165	A0A075B5R9	Immunoglobulin heavy v	Ighv14-3	585605	108600
166	G3X9D6	Apolipoprotein N GN=Ap	Apon	580625	269476
167	Q00898	Alpha-1-antitrypsin 1-5 G	Serpina1e	569606	63250
168	A0A140T8M5	Immunoglobulin kappa v	Igkv6-15	563449	239768
169	Q09PK2	Retroviral-like aspartic p	Asprv1	527975	185976
170	P01656	Ig kappa chain V-III regio	0	519142	226223
171	P11859	Angiotensinogen GN=Agt	Agt	481203	111830
172	A0A140T8P6	Immunoglobulin kappa v	Igkv12-46	469084	242003
173	P99026	Proteasome subunit beta	Psmb4	427152	260488
174	A0A140T8N0	Immunoglobulin kappa c	Igkv9-120	378529	152654
175	A0A0B4J1I1	Immunoglobulin kappa v	Igkv16-104	370721	114811
176	Q61268	Apolipoprotein C-IV GN=	Apoc4	351942	86004
177	Q62266	Cornifin-A GN=Sprr1a	Sprr1a	344453	591303
178	P01637	Ig kappa chain V-V region	0	334002	24338
179	Q61176	Arginase-1 GN=Arg1	Arg1	329251	306276
180	A0A0G2JFZ3	Immunoglobulin kappa c	Igkv19-93	326910	85124
181	O88783	Coagulation factor V GN=	F5	309846	10213
182	O35245	Polycystin-2 GN=Pkd2	Pkd2	309218	185105
183	Q8C872	Transferrin receptor prot	Tfrc	278896	87747
184	L7N274	Vomeronasal 2, receptor	Vmn2r95	270842	8915
185	A0A0G2JGN3	Immunoglobulin heavy v	Ighv1-78	269694	132310
186	Q8CAV0	Endoribonuclease YbeY G	Ybey	267585	94635
187	P33587	Vitamin K-dependent pr	Proc	248416	62519
188	P00920	Carbonic anhydrase 2 GN	Ca2	242066	19580
189	Q8N9S3	Activator of 90 kDa heat	Ahsa2	238886	2229

190	Q3UEP5	Sulfotransferase GN=Sult	Sult2a2	223925	19726
191	A0A075B5M7	Immunoglobulin kappa v	Igkv5-39	191582	47685
192	Q05816	Fatty acid-binding prote	Fabp5	183590	206251
193	Q9R1P0	Proteasome subunit alph	Psma4	139763	92731
194	A0A140T8N9	Immunoglobulin kappa v	Igkv6-32	130290	9541
195	A0A494BB08	Coiled-coil domain-cont	Ccdc88b	129316	122017
196	A6PWX9	Ubiquinol-cytochrome-c	Uqcc1	120007	18490
197	P01660	Ig kappa chain V-III regio	0	112414	31256
198	A0A0U1RPT5	Fructose-bisphosphate a	Aldoa	111451	98492
199	A2RT60	Serine protease HTRA4 G	Htra4	108156	14116
200	A0A075B5T2	Immunoglobulin heavy v	Ighv6-3	103441	20274
201	Q03734	Serine protease inhibitor	Serpina3m	90329	7377
202	D3Z5G7	Carboxylic ester hydrolas	Ces1b	87910	9080
203	Q8CIZ8	von Willebrand factor GN	Vwf	81039	14146
204	O35930	Platelet glycoprotein Ib a	Gp1ba	74626	40308
205	D3YTU6	Potassium channel, subfa	Kcnt2	49932	9878
206	A0A0B4J1H8	Immunoglobulin kappa v	Igkv1-133	45451	6282

Supplementary Table 5 : Absolute protein amount from each protein identified on the corona of nanoparticles preferential to hepatocytes (NH-1)

	Accession	Description	Gene	Intensity	STD
1	P07724	Albumin GN=/	Alb	9867188814	81671148
2	Q921I1	Serotransferri	Tf	968176195	76499244
3	Q61838	Pregnancy zor	Pzp	657270685	54897281
4	P01027	Complement (	C3	614413103	38736035
5	Q00623	Apolipoprote	Apoa1	594647373	102623269
6	Q00897	Alpha-1-antitr	Serpina1d	432195978	2036418
7	E9PV24	Fibrinogen alp	Fga	361949685	18741923
8	P28665	Murinoglobul	Mug1	298230815	37574911
9	A0A0R4J0I1	Serine proteas	Serpina3k	272167742	3965100
10	Q8K0E8	Fibrinogen be	Fgb	261234261	10034250
11	P06728	Apolipoprote	Apoa4	259818527	60996316
12	P06909	Complement f	Cfh	251844551	51136105
13	P23953	Carboxylester	Ces1c	206290217	15014848
14	A0A075B5P6	Immunoglobu	Ighm	199210569	42620194
15	P08226	Apolipoprote	Apoe	163958860	20220584
16	P11276	Fibronectin G	Fn1	148429215	2680242
17	Q8VCM7	Fibrinogen ga	Fgg	146238343	9890608
18	O08677	Kininogen-1 G	Kng1	135475306	6353932
19	G3X8Q5	Ceruloplasmir	Cp	127834019	13211990
20	P29699	Alpha-2-HS-gl	Ahsg	125701723	9658398
21	Q91X72	Hemopexin G	Hpx	107969223	11626875
22	P20918	Plasminogen (	Plg	92525843	5397232
23	Q06890	Clusterin GN=	Clu	86670234	15332284
24	P19221	Prothrombin	F2	86193604	4876317
25	P01837	Immunoglobu	Igkc	82183401	2807956
26	P13020	Gelsolin GN=G	Gsn	71070327	6797542
27	B7ZCR0	Uridine-cytidi	Uckl1	69755732	8042889
28	O89020	Afamin GN=Af	Afm	65618364	3194856
29	P07309	Transthyretin	Ttr	60829848	3013498
30	E9Q5L2	Inter alpha-try	Itih4	59435634	1796124
31	Q61703	Inter-alpha-try	Itih2	59261042	4639224
32	A8DUK4	Beta-globin G	Hbb-bs	59110124	4993830
33	P01029	Complement (	C4b	57481038	8925236
34	A0A0R4J0X5	Alpha-1-antitr	Serpina1c	53288351	454854
35	F8WJ05	Inter-alpha-try	Itih1	52667459	6643337
36	P32261	Antithrombin	Serpinc1	46649553	1211963
37	P21614	Vitamin D-bin	Gc	42660950	3965142
38	Q9QUK9	TESP4 GN=Try	Try5	42193520	1732260
39	P06684	Complement (	C5	36259363	7420345
40	P04186	Complement f	Cfb	34783450	3071104
41	P09813	Apolipoprote	Apoa2	30505324	4379105
42	A0A0A6YVP0	Immunoglobu	Ighg2b	27827254	836514
43	P03953	Complement f	Cfd	26566179	6107273
44	P97290	Plasma protea	Serping1	26008871	2173116
45	P29788	Vitronectin G	Vtn	25440276	3018398

46	Q07456	Protein AMBP	Ambp	24966878	2091488
47	Q61129	Complement f	Cfi	23629677	873599
48	Q01339	Beta-2-glycop	Apoh	21619509	858395
49	Q61247	Alpha-2-antip	Serpinf2	21586570	1696282
50	Q91VB8	Alpha globin 1	Hba-a1	21349827	1665468
51	P52430	Serum paraox	Pon1	18371410	1731941
52	Q9DBD0	Inhibitor of ca	Ica	18208267	1514186
53	A0A338P699	Kininogen 2 (I	Kng2	17263244	517727
54	A0A0R4J039	Histidine-rich	Hrg	17119479	1821500
55	Q60590	Alpha-1-acid g	Orm1	17050007	2284793
56	P22599	Alpha-1-antitr	Serpina1b	17012956	170403
57	A0A1Y7VJN6	Immunoglobu	Ighg3	15918975	1718622
58	E9QP56	Apolipoprote	Apoc3	15629057	132965
59	A0A0A6YY53	Immunoglobu	Ighg2c	15147219	2003033
60	Q9Z1R3	Apolipoprote	Apom	15143798	27276
61	E9Q1Y3	Apolipoprote	Apob	14856768	309257
62	A0A0A6YWR2	Ig gamma-1 ch	Ighg1	14555499	597309
63	A0A2I3BRQ3	Inter-alpha-try	Itih3	14284201	1194085
64	P01646	Ig kappa chain	0	13744815	1243438
65	P01898	H-2 class I hist	H2-Q10	13260846	1001635
66	Q19LI2	Alpha-1B-glyc	A1bg	12358411	636762
67	P41317	Mannose-bind	Mbl2	12300487	1488434
68	Q8VCU2	Glycosyl-phos	Gpld1	11990240	567221
69	P46412	Glutathione p	Gpx3	11709181	216518
70	P26262	Plasma kallikr	Klkb1	11456646	1210549
71	P34928	Apolipoprote	Apoc1	11304270	311026
72	Q02257	Junction plak	Jup	11078393	3248216
73	P42703	Leukemia inhi	Lifr	10784395	1016310
74	Q9DBB9	Carboxypeptid	Cpn2	9820779	302524
75	A0A075B5V1	Immunoglobu	Ighv1-31	9610911	1275952
76	P11680	Properdin GN	Cfp	8857608	294431
77	Q64726	Zinc-alpha-2-g	Azgp1	8674959	179477
78	O88947	Coagulation fa	F10	8604578	191556
79	P01723	Ig lambda-1 ch	0	8466016	1088075
80	Q80YC5	Coagulation fa	F12	8280360	1102229
81	A0A075B5N7	Immunoglobu	Igkv6-13	7966733	664579
82	Q6YJU1	Fetuin-B GN=F	Fetub	7784807	29943
83	Q06770	Corticosteroid	Serpina6	7055199	209381
84	A0A140T8P7	Immunoglobu	Igkv8-21	6592613	572933
85	A0A0A6YXW6	Immunoglobu	Igha	6543825	26539
86	Q01279	Epidermal gro	Egfr	6101057	320369
87	E9Q6I2	Interleukin-1	Il1rap	5935621	259231
88	Q9QWK4	CD5 antigen-li	Cd5l	5930484	840469
89	P49182	Heparin cofac	Serpind1	5731292	707156
90	P39039	Mannose-bind	Mbl1	5534956	262156
91	P04940	Ig kappa chain	0	5438223	1353656
92	P01679	Ig kappa chain	0	5285671	712754
93	P14847	C-reactive pro	Crp	5171366	839238

94	O70165	Ficolin-1 GN=	Fcn1	4616864	277359
95	P70274	Selenoprotein	Selenop	3778078	101650
96	P60710	Actin, cytopla	Actb	3691424	800635
97	A2A998	Complement	C8a	3683721	101382
98	Q8BH61	Coagulation fa	F13a1	3678967	353547
99	P51885	Lumican GN=L	Lum	3676300	320304
100	Q8CG14	Complement	C1sa	3301900	499474
101	Q9JJN5	Carboxypeptid	Cpn1	3295934	53752
102	P50446	Keratin, type I	Krt6a	3285112	1679472
103	Q9D7S9	Charged multi	Chmp5	3280850	38629
104	P31532	Serum amyloi	Saa4	3182998	38453
105	Q60994	Adiponectin G	Adipoq	3152869	177944
106	Q08879	Fibulin-1 GN=	Fbln1	3006537	33104
107	Q8BY71	Histone acety	Hat1	2983387	850012
108	A0A0G2JEU7	Immunoglobu	Ighv1-82	2937600	616269
109	F8WI14	Extracellular r	Ecm1	2901146	206828
110	Q9CPN7	RIKEN cDNA 1	1810009J06Ri	2665438	90014
111	Q8CJ96	Ras associatio	Rassf8	2600072	234116
112	P01657	Ig kappa chair	0	2518278	289697
113	Q02105	Complement	C1qc	2514056	260379
114	A0A075B5Y4	Immunoglobu	Ighv1-81	2502675	248567
115	Q9Z1T2	Thrombospor	Thbs4	2288397	222856
116	Q4FZE8	Major urinary	Mup22	2261543	173629
117	A0A1B0GSK1	Ufm1-specific	Ufsp2	2254189	279531
118	Q91XL1	Leucine-rich H	Lrg1	2244207	55404
119	A0A140T8N3	Immunoglobu	Igkv13-84	2231565	206306
120	A0A2R8VHR3	Mannan-bindi	Masp1	2185852	176534
121	P01887	Beta-2-microg	B2m	2169286	33224
122	P14106	Complement	C1qb	2038181	104232
123	G3X8T9	Serine proteas	Serpina3n	1867788	22649
124	Q00724	Retinol-bindi	Rbp4	1863003	9225
125	O35608	Angiopoietin-	Angpt2	1717100	130742
126	Q8BYZ7	Engulfment ar	Elmo3	1673363	26437
127	P51910	Apolipoprote	Apod	1663797	174912
128	P08607	C4b-binding p	C4bpa	1658386	343259
129	Q9DAC2	Complement	C8g	1583888	125953
130	Q8R121	Protein Z-dep	Serpina10	1560827	125750
131	Q07968	Coagulation fa	F13b	1547891	163304
132	A0A140T8M2	Immunoglobu	Igkv12-44	1473843	240891
133	A0A140T8N1	Immunoglobu	Igkv1-88	1440852	122913
134	A0A140T8Q3	Immunoglobu	Igkv6-17	1438248	50718
135	P16301	Phosphatidylc	Lcat	1404067	53445
136	A0A140T8P5	Immunoglobu	Igkv8-24	1378046	171235
137	P01665	Ig kappa chair	0	1334354	146967
138	Q8BND5	Sulfhydryl oxi	Qsox1	1295900	93249
139	P12246	Serum amyloi	Apcs	1220634	1999
140	Q8CG16	Complement	C1ra	1182092	157693
141	Q5SX59	2-phospho-D-	Eno3	1161207	262753

142	Q08761	Vitamin K-dep	Pros1	1148342	126434
143	P98086	Complement (	C1qa	1089001	277543
144	P01592	Immunoglobu	Jchain	1041305	86728
145	A0A1D5RLD8	Glyceraldehyd	Gm10358	988231	327024
146	P06683	Complement (	C9	986380	35059
147	P04104	Keratin, type I	Krt1	984660	92228
148	Q9CPN9	RIKEN cDNA 2	210010C04Ri	931998	255300
149	Q05020	Apolipoprote	Apoc2	885018	60219
150	E9Q5F6	Polyubiquitin	Ubc	870103	292242
151	A0A0R4J0S2	Insulin-like gr	Igfals	869762	101201
152	A0A075B5N9	Immunoglobu	Igkv3-7	862635	17697
153	P70663	SPARC-like pro	Sparcl1	838303	32766
154	Q9D5U0	Lysophosphat	Lpcat2b	830359	19420
155	A0A0B4J1J6	Immunoglobu	Ighv10-1	816568	60397
156	E9PUM5	Predicted gen	Gm4788	786086	46873
157	A0A571BF69	Maltase-gluco	Mgam	742469	37183
158	A0A3Q4EC30	Peptidase inh	Pi16	731972	56765
159	Q792Z1	Trypsin 10 GN	Try10	685939	47799
160	A0A075B5R9	Immunoglobu	Ighv14-3	684215	49937
161	G3X9D6	Apolipoprote	Apon	679676	45314
162	A0A140T8M5	Immunoglobu	Igkv6-15	613413	128754
163	S4R2K3	Protein-L-isoa	Pcmtd1	588956	147615
164	E0CZ49	Mitochondria	Trmu	587300	32421
165	Q00898	Alpha-1-antitr	Serpina1e	564091	12526
166	A0A0G2JFA8	Immunoglobu	Igkv17-121	541101	90533
167	P11859	Angiotensinog	Agt	524444	26596
168	O88783	Coagulation fa	F5	508435	48686
169	A0A075B677	Immunoglobu	Igkv4-53	460138	162202
170	A0A140T8P6	Immunoglobu	Igkv12-46	446544	49205
171	Q09PK2	Retroviral-like	Asprv1	433580	90783
172	P01656	Ig kappa chain	0	409364	49895
173	P01637	Ig kappa chain	0	405104	34350
174	A0A2I3BRS7	Krueppel-like	Klf10	396729	8882
175	Q61176	Arginase-1 GN	Arg1	392080	21333
176	A0A140T8N0	Immunoglobu	Igkv9-120	378296	1081
177	A0A0B4J1I1	Immunoglobu	Igkv16-104	376663	49448
178	A0A0G2JFZ3	Immunoglobu	Igkv19-93	374794	6241
179	Q61268	Apolipoprote	Apoc4	332439	35567
180	Q8CAV0	Endoribonucl	Ybey	330067	12855
181	P99026	Proteasome su	Psmb4	318113	67697
182	O35245	Polycystin-2 G	Pkd2	282004	19015
183	P33587	Vitamin K-dep	Proc	279800	18394
184	L7N274	Vomeronasal 2	Vmn2r95	272571	396
185	Q8N9S3	Activator of 9	Ahsa2	269300	7628
186	Q8C872	Transferrin rec	Tfrc	269167	18684
187	P00920	Carbonic anhy	Ca2	267352	10181
188	A0A0G2JGN3	Immunoglobu	Ighv1-78	215260	26119
189	A0A075B5M7	Immunoglobu	Igkv5-39	193894	9573

190	A0A140T8N9	Immunoglobu	Igkv6-32	146641	20193
191	Q3UEP5	Sulfotransfera	Sult2a2	142847	4776
192	Q62266	Cornifin-A GN	Sprr1a	120796	25588
193	A6PWX9	Ubiquinol-cyt	Uqcc1	119983	6563
194	Q05816	Fatty acid-bin	Fabp5	116102	8092
195	P01660	Ig kappa chain	0	114380	6116
196	D3Z5G7	Carboxylic est	Ces1b	104333	14590
197	A0A494BB08	Coiled-coil do	Ccdc88b	103400	23593
198	Q8CIZ8	von Willebran	Vwf	94315	12949
199	Q03734	Serine proteas	Serpina3m	91234	8682
200	A2RT60	Serine proteas	Htra4	85781	6230
201	A0A075B5T2	Immunoglobu	Ighv6-3	83010	1320
202	Q9R1P0	Proteasome su	Psma4	79336	14837
203	A0A0U1RPT5	Fructose-bisp	Aldoa	77423	18559
204	O35930	Platelet glyco	Gp1ba	75882	19437
205	D3YTU6	Potassium cha	Kcnt2	51816	4089
206	A0A0B4J1H8	Immunoglobu	Igkv1-133	50623	3423
